# *Hyalomma marginatum* in Europe: The Past, Current Status, and Future Challenges—A Systematic Review

**DOI:** 10.1155/tbed/7771431

**Published:** 2025-07-29

**Authors:** Seyma S. Celina, Jiří Černý

**Affiliations:** Center for Infectious Animal Diseases, Faculty of Tropical AgriSciences, Czech University of Life Sciences Prague, Prague, Czech Republic

**Keywords:** Crimean–Congo hemorrhagic fever virus, *Hyalomma marginatum*, migratory birds, *Rickettsia*, surveillance, tick distribution

## Abstract

*Hyalomma marginatum* is a prominent tick vector responsible for transmitting various pathogens, including the Crimean–Congo hemorrhagic fever virus (CCHFV), across Europe. This systematic review consolidates findings from 144 publications regarding the geographical distribution of *H. marginatum* and its associated pathogens. Significant populations have been identified primarily in Southern Europe and Balkan Peninsula, indicating a concerning trend. Additionally, climate change and migratory bird movements may facilitate its further dispersal, potentially leading to the establishment of *H. marginatum* in transalpine regions. Consequently, robust monitoring and surveillance strategies are essential to mitigate the public health and livestock threats posed by *Hyalomma*-borne diseases. Raising awareness and implementing preventive measures will be crucial in addressing the challenges associated with this tick vector.

## 1. Introduction

Ticks (Acari: Ixodidae) are the most significant vectors of arthropod-borne diseases in Europe, transmitting numerous pathogens to humans, livestock, and wildlife [1, 2]. The genus *Hyalomma* are two-host species, where both subadult developmental stages feed on the same host, typically small mammals such as rodents, hares, or ground-feeding birds. In contrast, adult ticks prefer to feed on larger mammals, including humans [3]. However, some species, such as *H. aegyptium*, follow a three-host life cycle, in which the larval, nymphal, and adult stages each feed on different host species. Large domestic mammals play a crucial role in maintaining high tick loads and increasing the risk of exposure to *Hyalomma*-borne pathogens due to their proximity to agricultural workers, either through crushing engorged ticks or via contact with infected blood during slaughter [4–6]. Migratory birds serve both as a blood meal source for immature *Hyalomma* ticks and plus introduce these ticks into new geographic regions [7].

*Hyalomma marginatum*, commonly known as the “Mediterranean *Hyalomma*,” has historically been referred to as *Hyalomma plumbeum* in some Russian and Eastern European literature. However, *Hyalomma plumbeum* is not an exclusive synonym for *H. marginatum*, but has also been used for other species, including *Hyalomma marginatum turanicum* [8, 9], *Hyalomma plumbeum turanicum* [10], *Hyalomma rufipes* [11], and *Hyalomma rufipes glabrum* [12].

Historically, *Hyalomma* (Euhyalomma) *marginatum* Koch, 1844 was recognized as a taxonomic complex comprising four subspecies: *Hyalomma* (E.) *marginatum marginatum* Koch, 1844; *Hyalomma* (E.) *marginatum rufipes* Koch, 1844; *Hyalomma* (E.) *marginatum turanicum* Pomerantzev, 1946; *Hyalomma* (E.) *marginatum isaaci* Sharif, 1928 [13]. Overtime, the taxonomy of the *H. marginatum* complex has undergone major revisions, leading to the reclassification of some of these taxa as distinct species. In 2008, Apanaskevich and Horak [14] redefined *H. rufipes*, *H. turanicum*, and *H. isaaci* as distinct species. Currently, the *Hyalomma marginatum* sensu lato (s.l.) complex includes five distinct species: *Hyalomma marginatum* (sensu stricto), *Hyalomma rufipes*, *Hyalomma isaaci*, *Hyalomma turanicum*, and *Hyalomma glabrum*, which are distributed across the Afrotropical, Palearctic, and Oriental regions [15].

*Hyalomma marginatum* ticks primarily inhabit steppe, savannah, and scrubland hills and valleys with a Mediterranean climate characterized by low to moderate levels of humidity and long, hot, and dry summers [16]. They are notably absent from European deciduous and mixed forests, which are more commonly inhabited by *Ixodes ricinus* [7].

*Hyalomma marginatum* is primarily found in North Africa, Southern Europe, and parts of Asia, whereas *H. rufipes* is mostly present in sub-Saharan Africa and the region around the Red Sea and has recently expanded its range into the Eastern Palearctic [14]. The distinction between *H. rufipes* and *H. marginatum* remains challenging due to their morphological similarities and overlapping ecological niches, as noted by the European Centre for Disease Prevention and Control (ECDC) [16]. This ambiguity can introduce bias in literature-based research, including this review, and we address this issue by noting potential outdated or inaccurate taxonomic classifications when relevant.

*Hyalomma marginatum* is of significant veterinary and public health importance due to its role in transmitting various tick-borne pathogens. This review specifically focuses on *H. marginatum*, which is the primary vector of Crimean–Congo hemorrhagic fever virus (CCHFV) in Europe, an emerging arbovirus with a human mortality rate of up to 40% [17–19]. *Hyalomma* ticks acquire the virus when feeding on the blood of infected mammals, although viremia in the mammalian host is transient. Unlike mammals, ticks act as natural reservoirs for CCHFV, remaining infected for several years. Humans, however, serve as dead-end hosts, with infections occurring through tick bites or direct contact with the blood or tissues of infected livestock or patients. The disease initially presents with nonspecific symptoms such as headaches, muscle pain, and fever, which can progress to severe hemorrhagic manifestations, including skin hemorrhages.

The endemic range of CCHFV closely matches the geographical distribution of *H. marginatum*. In addition to CCHFV, *H. marginatum* also transmits a range of other pathogens, including bacterial and protozoal agents such as *Anaplasma*, *Rickettsia*, *Babesia*, and *Theileria* species, which can cause diseases in both humans and animals [20–22].

Among *Rickettsia* spp., *Hyalomma* ticks are competent vectors of the spotted fever group (SFG). The SFG *Rickettsia* are responsible for spotted fever, a condition marked by papular exanthems, eschars, and skin lesions, accompanied by high fever, muscle and joint pain, headaches, and photophobia. Several pathogens within this group, including *Rickettsia aeschlimannii*, *R. africae*, *R. raoultii*, and *R. sibirica mongolotimonae*, are associated with human diseases such as spotted fever rickettsiosis, African tick bite fever (ATBF), tick-borne lymphadenopathy (TIBOLA), and lymphangitis-associated rickettsiosis (LAR), all of which have been detected in *H. marginatum* [23–29].

This review provides a comprehensive assessment of the distribution of *H. marginatum* and its associated pathogens across various regions of Europe. By integrating information from multiple sources, this study aims to inform vector surveillance programs and public health interventions in regions at risk for Crimean–Congo hemorrhagic fever (CCHF) and other *Hyalomma*-borne diseases.

## 2. Materials and Methods

### 2.1. Study Design and Search Strategy

This systematic review was conducted in accordance with the Preferred Reporting Items for Systematic Reviews and Meta-Analyses (PRISMA) guidelines [30]. We reviewed published studies reporting occurrence records of *H. marginatum* and *H. rufipes* in Europe. The systematic searches were conducted on 16 June, 2023 in two major databases: Scopus, a multidisciplinary database, and PubMed, a primary biomedical sciences database. The search was limited to English-language publications with no restrictions on the year of publication. We used the following Boolean operators and search terms: “(*Hyalomma marginatum*) OR (*Hyalomma rufipes*).” Titles and abstracts were screened and relevant full-text articles were retrieved via library resources and online platforms.

### 2.2. Selection Criteria and Outcome Measures

The inclusion criteria for articles involved the documented occurrences of *H. marginatum* across the European continent. Eligible studies described the collection of *H. marginatum* from vegetation, animals, or humans. Studies reporting the occurrence of *H. marginatum* in Europe, even if they lacked detailed information on host, location within the country, or tick counts, were also included. Additionally, studies that detected pathogens in *H. marginatum* ticks collected in Europe were considered. Given the complexity of *Hyalomma* species taxonomy and the challenges distinguishing *H. marginatum* from *H. rufipes*, studies that reported on either species or the *H. marginatum* s.l. complex in Europe were included to minimize potential bias; these results are presented separately. We excluded studies focused on laboratory-maintained tick populations, experimental investigations, tick behavior, systematics, insecticide resistance, or those on occurrences of *H. marginatum* or *H. rufipes* outside Europe. Publications in languages other than English and those for which the full original article could not be obtained were also excluded.

### 2.3. Data Extraction and Analysis

The extracted data were organized into the following categories (Table S1):

• Main characteristics of studies: This included information such as article ID, publication year, authors, and journal of publication.
• Tick-related information: Details about the ticks, including species, sex, developmental stage, collection date, identification methods (morphological or molecular), total number of ticks collected, the number subjected to pathogen detection, the number of positive ticks, and pathogen prevalence.
• Pathogen-related information: This covered the pathogens under investigation, diagnostic tests used, and species detected. Information about the number of pathogen species was also recorded.
• Host-related information: Data on host species, including both common and scientific names, the number of hosts screened, and the number of hosts carrying ticks.
• Geographical context: This included study location details, such as country, province, and, where available, GPS coordinates of the study sites.

The extracted data were used to generate maps illustrating the distribution and prevalence of *H. marginatum* and its associated pathogens across Europe. These maps were created using ArcGIS Pro 3.1.2.

## 3. General Aspects of *Hyalomma marginatum* Biology in Europe

### 3.1. Bibliometric Data

**Figure 1:**
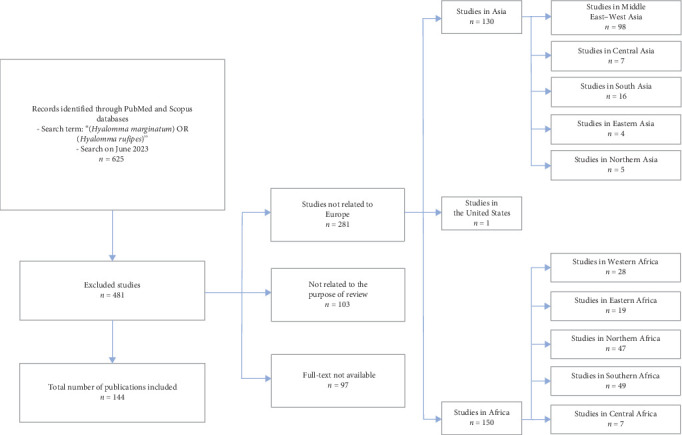
Systematic literature review methodology—PRISMA flow diagram.

This systematic review analyzed a total of 144 publications (Figure 1). Of these, 128 articles specifically focused on the presence of *H. marginatum* in Europe. Among these, 15 publications also reported the co-occurrence of *H. marginatum* with other members of the *H. marginatum* s.l. complex, including *H. rufipes*, *H. marginatum turanicum*, and references to broader taxonomic labels such as the *H. marginatum* complex, *H. marginatum* s.l., and *Hyalomma* spp. In some cases, the term *H. marginatum* sensu stricto was used to differentiate it from other species in the complex. Additionally, 11 articles focused exclusively on *H. rufipes*, two on the *H. marginatum* complex, two on *H. marginatum* s.l., and one article reported only *Hyalomma* spp.

Throughout this manuscript, unless otherwise indicated, *H. marginatum* refers to *H. marginatum* sensu stricto as a distinct species.

### 3.2. Geographic Distribution

The continent was divided into six regions: the Balkan Peninsula, Central, Eastern, Northern, Southern, and Western Europe. Southern Europe recorded the highest number of *H. marginatum* occurrences, with Italy (*n* = 36), Malta (*n* = 1), Portugal (*n* = 8), and Spain (*n* = 17) contributing a total of 62 articles, representing 48.4% of the total publications reporting exclusively on *H. marginatum* occurrences (Table S1). The results also indicated the presence of *H. marginatum* in the Balkans, with varying numbers of publications from each country: Albania (*n* = 1), Bosnia (*n* = 3), Bulgaria (*n* = 4), Croatia (*n* = 2), Greece (*n* = 6), Kosovo (*n* = 3), and Romania (*n* = 8). In Western Europe, most reports came from France (*n* = 14), particularly from its Mediterranean regions, with the United Kingdom (*n* = 3) and the Netherlands (*n* = 1) being the other countries in this region to report *Hyalomma* tick occurrences.

Central and Northern Europe had fewer publications on *H. marginatum*, with records from Austria (*n* = 1), Czechia (*n* = 1), Germany (*n* = 4), Hungary (*n* = 3), Poland (*n* = 2), Denmark (*n* = 1), Finland (*n* = 1), Norway (*n* = 1), and Sweden (*n* = 2), being the only countries reporting its occurrence. Similarly, Eastern Europe had limited publications on *H. marginatum*, with reports from Moldova (*n* = 1), Russia (*n* = 3), and Ukraine (*n* = 2); however, additional records from Russia were excluded from this analysis due to their availability only in Russian.

In contrast, 31 articles reported the presence of *Hyalomma* ticks without species-level identification, referring to them as *Hyalomma* spp., the *H. marginatum* complex, or *H. marginatum* s.l. Additionally, some studies specifically identified *H. rufipes* and *H. marginatum turanicum*. These studies covered Czechia (*n* = 4), France (*n* = 1), Germany (*n* = 2), Greece (*n* = 7), Hungary (*n* = 4), Italy (*n* = 12), Malta (*n* = 2), the Netherlands (*n* = 1), Norway (*n* = 1), Slovakia (*n* = 1), Spain (*n* = 3), Sweden (*n* = 1), and the United Kingdom (*n* = 1).

The findings of this review, based on the content of the analyzed studies rather than just the number of publications, indicate that *H. marginatum* is primarily established in the Mediterranean region of Europe. In contrast, sporadic occurrences have been documented in transalpine areas. Countries such as Austria [31], Czechia [32], Finland [33], Germany [34–36], Hungary [37, 38], the Netherlands [39], Poland [40, 41], Sweden [26], and the United Kingdom [42, 43] have reported isolated cases, often linked to imported animals, humans, or migratory birds. No evidence currently supports the presence of established populations in these regions.

It is important to note that the number of publications does not necessarily reflect true distribution, but rather the intensity of surveillance and research efforts. Regions with few or no reports may still harbor *H. marginatum* populations that remain undocumented due to limited sampling or reporting.

### 3.3. Preferred Hosts

**Figure 2:**
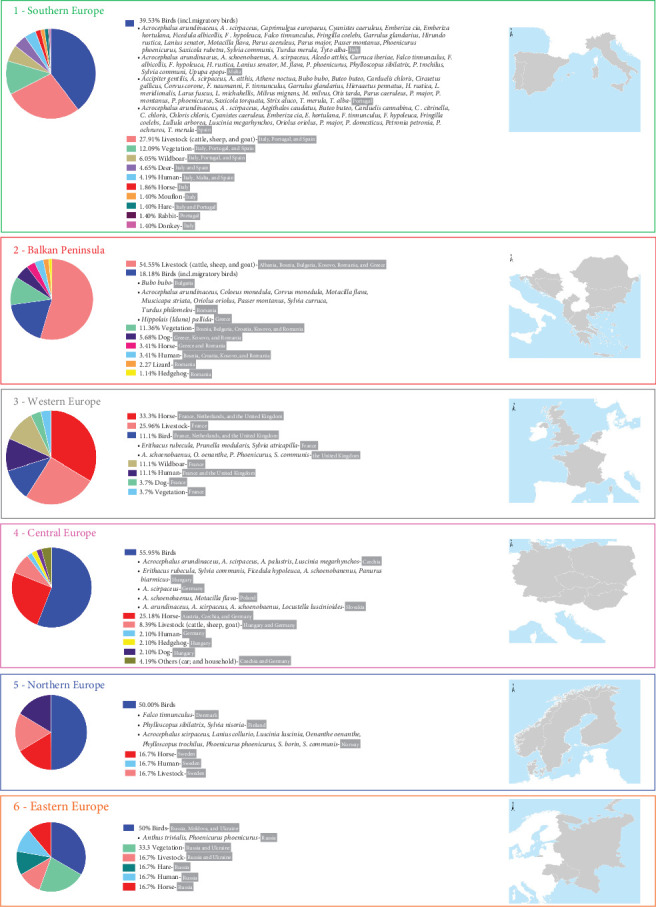
Distribution of sources of *Hyalomma marginatum* collection across Europe.

The study also assessed host associations of *H. marginatum* across Europe, identifying cattle and birds as the most frequently reported hosts across all regions (Figure 2). In the Balkans, *H. marginatum* was also collected from vegetation (in Bulgaria, Croatia, Kosovo, and Romania), horses (in Greece and Romania), dogs (in Greece, Kosovo, and Romania), and, less frequently, from humans (in Bosnia, Croatia, Kosovo, and Romania) [44–69]. The tick species was also recorded on lizards and hedgehogs in Romania [44].

In Western Europe, horses were the primary hosts, followed by cattle. In the Netherlands, all records involved horses, except for a single *H. rufipes* from a bird [39]. In France, *H. marginatum* was found on a wider variety of hosts, including cattle, horses, birds, dogs, wild boars, humans, and vegetation [28, 70–81]. In the United Kingdom, *H. marginatum* were collected from birds, horses, and a human [42, 43, 82].

In Central and Northern Europe, *H. marginatum* was primarily collected from birds, but also from horses (in Austria, Czechia, Germany, and Sweden), livestock (in Hungary, Germany, and Sweden), humans (in Germany and Sweden), and dogs and hedgehogs (in Hungary) [26, 31, 32, 34–38, 83–86]. In Eastern Europe, birds were again the most common source (in Russia, Moldova, and Ukraine), but ticks were also found on hares (in Russia), livestock (in Russia and Ukraine), humans (in Russia), and horses (in Russia) as well as from vegetation (in Russia and Ukraine) [87–90].

In Southern Europe, *H. marginatum* was mainly collected from cattle and birds, but also from vegetation (in Italy, Portugal, and Spain), deer (in Italy and Spain), wild boar (in Italy, Portugal, and Spain), humans (in Italy, Malta, and Spain), hares (in Italy and Portugal), rabbits (in Portugal), donkeys, mouflons, and horses (in Italy; Figure 2) [23, 25, 27, 91–145].

### 3.4. Vector Capacity

**Figure 3:**
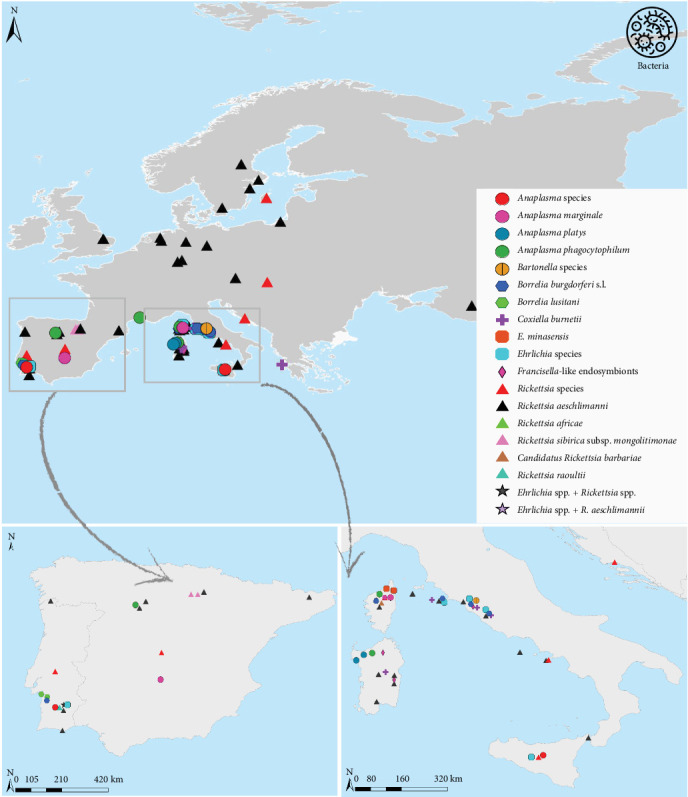
Distribution of bacteria detection in *Hyalomma marginatum* ticks across Europe and close-ups of Southern Europe (Italy, Portugal, and Spain), to provide additional details to bacteria species detected in *Hyalomma marginatum* ticks in the region.

**Figure 4:**
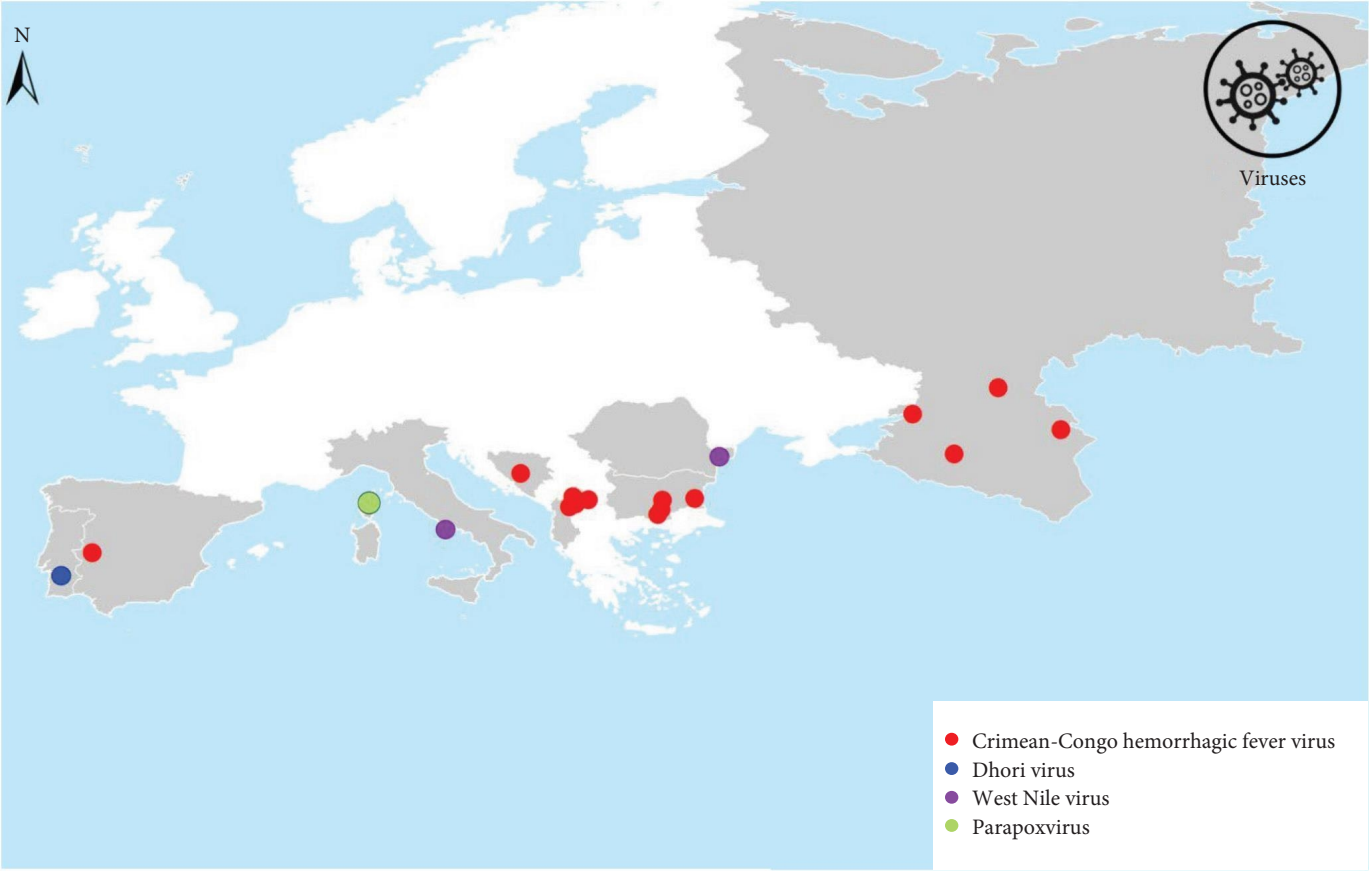
Distribution of virus detection in *Hyalomma marginatum* ticks across Europe.

**Figure 5:**
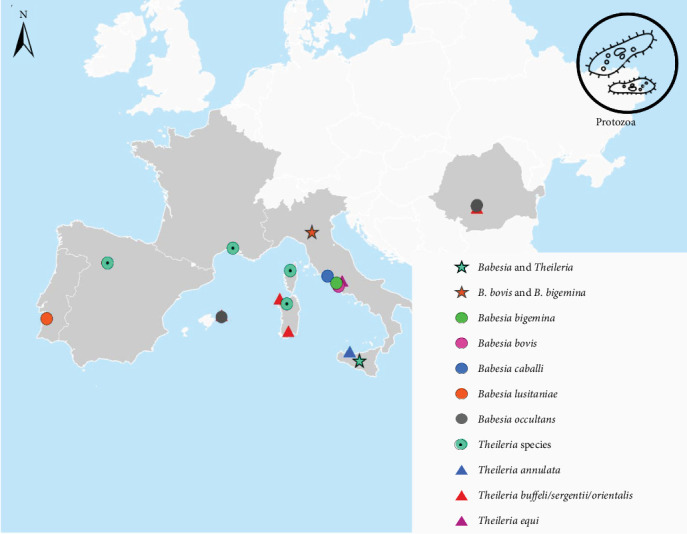
Distribution of protozoa detection in *Hyalomma marginatum* ticks across Europe.

Of the 128 publications reviewed, 36.7% focused on the detection of bacterial pathogens in *H. marginatum* (Figure 3), 10.9% reported viral pathogens (Figure 4), and 7% protozoan pathogens (Figure 5). In total, 24 pathogens were identified in *H. marginatum* specimens collected across Europe. These pathogens were classified into seven bacterial genera, four viral genera, and two protozoan genera (Table 1).

*Hyalomma marginatum* is a known vector of several viruses—including Thogoto, Dhori, and CCHFV—as well as bacterial and protozoal agents such as *Rickettsia*, *Anaplasma*, *Babesia*, and *Theileria*. In Europe, CCHFV and *Rickettsia* species are the most frequently reported. CCHFV, a zoonotic virus of the *Orthonairovirus* genus (family *Nairoviridae*), is one of the most widespread tick-borne viruses affecting humans [146]. While *Hyalomma* ticks are the main vectors of CCHFV, other tick species, such as *Rhipicephalus* and *Dermacentor*, may also contribute to the virus's life cycle [147–150].

*Rickettsia* species, particularly *R. aeschlimannii*, are frequently found in *H. marginatum* populations. SFG rickettsiae, transmitted by *Hyalomma* ticks, cause a range of febrile illnesses in humans, often characterized by rash, eschars, and systemic symptoms. The presence of *R. aeschlimannii*, *R. africae*, *R. raoultii*, and *R. sibirica mongolotimonae* in European *H. marginatum* populations highlights their significance in emerging zoonotic disease ecology.

This review further demonstrates that *H. marginatum* frequently harbors multiple pathogens at notable prevalence rates. To offer a comprehensive overview of the prevalence of CCHFV and *Rickettsia* species in *H. marginatum* across Europe, Tables 2 and 3 summarize key data, including collection dates, locations, sources of tick collection, pathogen prevalence, and the detection methods employed, as reported in the literature.

## 4. Overview of *Hyalomma marginatum* Distribution and Its Role as a Vector for Pathogens in Main European Regions

The following subsections present a structured overview of *H. marginatum* distribution across various European regions, organized as follows:

• Regions are presented in order of the number of publications and their relevance to *H. marginatum* distribution.
• Within each region, countries are listed alphabetically to enhance readability.
• For some countries (France, Italy, and Spain), information is organized by subregions.
• Information for all countries and subregions is presented chronologically.

### 4.1. *Hyalomma marginatum* Distribution in Southern Europe

#### 4.1.1. Italy

Italy lies along a major migratory route for birds carrying *H. marginatum* larvae and nymphs. The tick was first recorded in Italy near Naples in 1973, discovered on a racehorse transported from Northern Italy [143]. Since then, the tick has been increasingly reported across various habitats and hosts, highlighting its ecological plasticity and expanding role as a vector.

*Sicily*, with its Mediterranean climate—dry summers and mild winters—is one of the primary hotspots for *H. marginatum* in Europe. The island consistently reports high burdens of tick-borne diseases in livestock. A survey conducted in 1998–1999 found *H. marginatum* to be the most common tick species on cattle, accounting for 27.1% of the total ticks, with some specimens testing positive for *Theileria annulata* and *T. buffeli/sergentii/orientalis* [141]. In a 2001–2002 survey, *Rickettsia aeschlimannii* was detected in two out of 48 *H. marginatum* ticks and *R. africae* was identified in one specimen [142]. A further survey conducted between 2002 and 2003 found that 207 out of 6208 ticks (3.3%) collected from cattle, sheep, and vegetation were *H. marginatum*. Among these, 9.9% tested positive for pathogens, including *Babesia/Theileria* (16.4%), *Rickettsia* spp. (4.5%), *Ehrlichia* spp. (3%), and *Anaplasma* spp. (1.5%) [131].

*In Southern Italy*, a retrospective study conducted on samples collected in 2000 found 24 *H. marginatum* (out of 744 ticks tested) to be positive for *Babesia*, *Theileria*, and *Anaplasma*, although no positive results were detected for certain pathogens [137]. *Hyalomma marginatum* has been identified on both wild and domesticated species, including dogs, livestock, and wildlife. In 2009, *H. marginatum* was found on vegetation and hares in a wildlife reserve, with 51 adults and 165 nymphs collected [134], as well as one additional adult female from a road-killed hare [139]. A separate survey recorded 109 *H. marginatum* specimens among 10,795 ticks collected from vegetation [135]. Another investigation in the Basilicata region identified 15 *H. marginatum* specimens [126]. In a study testing 123 ticks from mammals and vegetation for *Rickettsia*, five ticks (4.1%) were identified as *H. marginatum*, including three from Corsican red deer and two from mouflon, with one mouflon tick testing positive for *R. aeschlimannii* [125]. A broader survey of 50,325 birds across 13 ringing sites in Southern and Central Italy (2013–2014) found that 0.22% were infested with *Hyalomma* ticks, suggesting the potential for dissemination of this tick via migratory birds [157]. Additionally, a study in Italy and Spain documented *H. marginatum* with dual symbiosis involving *Francisella*-like and *Midichloria* endosymbionts, marking the first such finding in ticks [144]. On Capri Island, located off the southwestern coast of Italy, a separate study conducted in 2014–2015 collected 575 ticks from migrating birds during migration, identifying *H. rufipes* (77.7%) and *H. marginatum* (4.2%) [158]. *Hyalomma marginatum* was detected on a variety of bird species, with *H. rufipes* being prevalent across multiple species. Pathogen screening revealed *Francisella* spp. in 76.7% (343/447) of *H. rufipes* and 75% (18/24) of *H. marginatum*, and SFGR in 61.5% (275/447) of *H. rufipes* and 50% (12/24) of *H. marginatum*. Further analysis confirmed the presence of *Francisella*, *Rickettsia*, and *Midichloria* in two *H. rufipes* ticks, reinforcing the health risks associated with *Hyalomma* ticks in Southern Europe [159].

*Sardinia* has also emerged as a key focus for *H. marginatum* surveillance. In 2007, 83 *H. marginatum marginatum* ticks were collected from horses, with 11 testing positive for *R. aeschlimannii* [140]. A separate study conducted during the same period reported 15 (1%) *H. marginatum* ticks on cattle, although no pathogens were detected [136]. Subsequent surveys have identified *H. marginatum* on cattle, dogs, and wild animals, with 8.3% testing positive for *R. aeschlimannii* in one instance [115]. *Hyalomma marginatum* was also found on wild donkeys in Sardinia, with 12 (1.9%) of 256 ticks identified on this host [120]. Chisu et al. [123] reported 14 *H. marginatum* out of 115 ticks collected from domestic and wild animals, with all ticks collected from common whitethroats (*Sylvia communis*) testing positive for *Anaplasma phagocytophilum* and *A. platys* (100%), while one specimen from a wild boar was positive for *A. platys* [123]. Between 2010 and 2015, a large-scale survey on 1619 animal ticks in Sardinia identified 45 *H. marginatum*, with 33.3% testing positive for *Rickettsia* spp.% and 4.4% for *Coxiella burnetii* [133]. Among humans, a survey of 42 ticks collected from 2012 to 2013 found two *H. marginatum* specimens, both testing positive for *R. aeschlimannii* (100%) [119]. Another survey identified 16 *H. marginatum* ticks among 185 ixodid ticks, with two specimens (12.5%) testing positive for *R. aeschlimannii* [154]. Additional pathogens detected in *H. marginatum* included *Francisella* spp. DNA, *T. buffeli/sergentii/orientalis*, and *C. burnetii*, emphasizing the zoonotic potential of *H. marginatum* ticks in the region as potential vectors of zoonotic pathogens [114, 117].

*In western Italy*, on Ventotene Island, a stopover site for migratory birds, the presence of exotic tick species and tick-borne pathogens was investigated by trapping birds [122]. *Hyalomma marginatum* was confirmed as the second most abundant tick species (2.3%, 10/433), following *H. rufipes* (82.6%, 366/443). Five *H. marginatum* ticks tested positive for *R. aeschlimannii*; one nymph collected from a European nightjar (*Caprimulgus europaeus*) and another from a western yellow wagtail (*Motacilla flava*) were positive for *Flavivirus*, with West Nile Virus (WNV) RNA identified in the latter [122]. Between 2017 and 2019, studies of migratory birds and vegetation reported *H. marginatum* in 4.3% of 2681 collected ticks [130]. Further, a survey of wild ungulates and their ectoparasites found a single *H. marginatum* on a wild boar, though no *Anaplasma* DNA was detected [116]. In a separate study conducted between 2017 and 2019, 2344 ticks were collected from 1079 birds. Two ticks identified as *H. marginatum* and *Hyalomma* spp. tested positive for WNV, while CCHFV RNA was detected in two immature *H. rufipes* ticks from Ventotene Island. Although *H. marginatum* has tested positive for WNV RNA, there is no evidence that it acts as a biological vector. WNV transmission primarily occurs via *Culex* mosquitoes and the presence of WNV in *Hyalomma* ticks is likely due to ingestion from infected hosts rather than active replication. Further studies are needed to confirm any potential vector competence.

*Hyalomma* ticks were prevalent on various migratory birds, with notable counts of 28 from golden orioles (*Oriolus oriolus*), 83 from wood warblers (*Phylloscopus* spp.), and 65 *H. rufipes* from common redstarts (*Phoenicurus phoenicurus*) and whinchats (*Saxicola rubetra*), among others from species like the icterine warbler (*Hippolais icterina*) and woodchat shrike (*Lanius senator*). These findings emphasize the role of migratory birds in introducing African ticks and zoonotic pathogens into Europe [145]. On Pianosa Island, 11 *H. marginatum* were collected from vegetation in 2006–2007, with two (18.2%) testing positive for *R. aeschlimannii* [124].

*In Central and Northern Italy*, *H. marginatum* has frequently been found on livestock. A 2005 survey reported infection rates of 4.5% for *Babesia bovis* and 2.7% for *B. bigemina* in *H. marginatum* ticks from cattle, while 1.8% of ticks collected from horses tested positive for *T. equi* [129]. Further studies revealed that 22.8% of *H. marginatum* ticks from cattle were infected with *Babesia*, with 27.6% exhibiting dual infections with *B. bigemina* and *B. bovis* [128]. In the spring of 2009, 386 ticks were collected from migratory birds on Capri and Antikythera islands, 369 of which were identified as *Hyalomma*, with nine identified as *H. rufipes* and one as *H. marginatum* [69]. Ornithological surveys in Central Italy in 2010–2011 collected 137 ticks from 41 birds across 17 species, all identified as nymphs within the *Hyalomma* genus [160]. Surveys conducted in Lazio in 2010–2011 found *H. marginatum* nymphs on migratory birds, with pathogens such as *R. aeschlimannii* (21.6%), *Ehrlichia* spp. (16.2%), C. *burnetii* (27.0%), and *Borrelia burgdorferi* sensu lato (s.l.) (73.0%) detected [27]. In the Maremma region of Lazio, Central Italy, a 2011–2012 study collected 154 ticks, identifying four as adult *H. marginatum* [132]. Between 2012 and 2013, additional surveys in Lazio and Tuscany found 10 *H. marginatum* ticks (8.8%), with one testing positive for *R. aeschlimannii* (10%) [121]. Another survey in Lazio collected 96 ticks from vegetation, 49 of which were identified as *H. marginatum*, with one testing positive for *Babesia caballi* (2%) [138]. Molecular screening of 255 tick samples, including 12 *H. marginatum* (5%), revealed positivity for *R. aeschlimannii* (66.7%), *Francisella*-like endosymbionts (66.7%), *B. burgdorferi* s.l. (50%), *Bartonella* spp. (25%), *C. burnetii* (33%), and *Ehrlichia* spp. (16%) [118]. In 2019, a study on Ponza Island collected 14 *H. rufipes* ticks from various birds, detecting *R. aeschlimannii* in six specimens, *Francisella*-like endosymbionts in all, and *B. burgdorferi* s.l. in four ticks [155].

In summary, Italy represents a critical zone for the establishment and spread of *H. marginatum* in Europe, due to its favorable Mediterranean climate, high livestock density, and role as a major migratory bird corridor. The species is well-established in southern regions and islands like Sicily and Sardinia, with consistent reports of pathogen detections—including *Rickettsia spp*., *C. burnetii*, *Babesia*, *Theileria*, and *Anaplasma*—from both domestic and wild hosts. These findings highlight Italy's importance in regional tick ecology and the need for sustained surveillance to monitor *Hyalomma*-borne disease risks.

#### 4.1.2. Malta

Reports confirm the presence of *H. marginatum* in Malta. A recent study identified this tick species among 113 ixodid ticks collected from various bird species. Notably, one was found on a common kingfisher (*Alcedo atthis*), another on a common kestrel (*Falco tinnunculus*), and one on a common whitethroat. Additionally, two *H. marginatum* ticks were identified on sedge warblers (*Acrocephalus schoenobaenus*) and two on willow warblers (*Phylloscopus trochilus*) [98]. Furthermore, *H. rufipes* was identified on a broader range of birds, including 14 ticks from great reed warblers (*Acrocephalus arundinaceus*) and three from Eurasian reed warblers (*Acrocephalus scirpaceus*). Other species, such as barn swallows (*Hirundo rustica*), woodchat shrikes, and yellow wagtails, were also found to carry *H. rufipes*. A total of four *Hyalomma* spp. ticks were collected from sedge warblers, wood warblers, and common whitethroats. A 2021 study also reported the presence of *H. rufipes* on a migrant human, though no evidence of CCHFV or other pathogens were detected in the patient [161]. Findings from Malta highlight the island's role as an important stopover site for migratory birds carrying *H. marginatum* and *H. rufipes*. Although the number of ticks detected remains low and no pathogens have been found in humans, the wide range of avian hosts suggests ongoing passive introduction. These observations support the need for continued surveillance to monitor the potential establishment and spread of *Hyalomma* ticks in the region.

#### 4.1.3. Portugal

*Hyalomma marginatum* is widely distributed across the westernmost country of continental Europe. It was first recorded in cattle in Vidigueira in 1971, with 177 adult male ticks collected; one pool of ticks tested positive for Dhori virus, with a 20% infection rate [96]. In 1985, two human cases of CCHFV seropositivity were reported in Southern Portugal [162]. A 1998 study recorded 27 *H. marginatum* ticks among 82 ticks collected from vegetation, with two (7.4%) testing positive for *B. lusitaniae* [97]. A nationwide survey in 2001 identified five *H. marginatum* ticks (6.6%) out of 76 total ticks collected, while in the Mafra and Grandola regions, 62 *H. marginatum* ticks (1.1%) were found among a total of 5459 ticks, with two testing positive for *B. burgdorferi* s.l. and two for *B. lusitaniae* [94].

Between 2002 and 2004, 118 *H. marginatum* ticks (77.6%) were collected from various bird species in Santo Andre Natural Reserve and Monsanto Forestal Park, with three testing positive for *R. aeschlimannii* [92]. From 2006 to 2009, a study conducted in a Safari Park in Alentejo collected 677 ticks, identifying eight as *H. marginatum* [93]. In an updated study by Santos-Silva et al. [95], *H. marginatum* was found to be the fourth most common tick in Portugal, with 6.1% of the total ticks collected, primarily from vegetation and ungulates. Immature stages were predominantly found on birds and hares. Between 2010 and 2011, four *H. marginatum* ticks were identified from a sample of 848 ticks collected in Tapada de Mafra National Park and 22 *H. marginatum* were found among 1122 ticks sampled from birds during the same period [91]. Pereira et al. [25] reported 176 *H. marginatum* ticks (29.7%) collected from various hosts, with positive results for *Anaplasma*, *Rickettsia* spp., *R. aeschlimannii*, *R. raoultii*, *Ehrlichia* spp., and mixed infections involving *Ehrlichia* and *Rickettsia* spp.

In Portugal, *H. marginatum* appears to be well-established across diverse habitats, with records spanning livestock, birds, wildlife, and vegetation. Pathogen detections over several decades—ranging from *Borrelia* spp. and *Rickettsia* spp. to *Anaplasma* and *Ehrlichia*—reflect both historical and ongoing public and veterinary health relevance. The presence of immature ticks on birds suggests continued passive dispersal, while detections in ungulates and vegetation confirm local maintenance.

#### 4.1.4. Spain

*Hyalomma marginatum* is widely distributed across various regions in the country.

*In Northern Spain*, a survey conducted in 1998, *H. marginatum marginatum*was scarcely found among 12,832 ticks collected from sheep [109]. In Castilla y León, a survey conducted between 1996 and 2002 identified 324 *H. marginatum* ticks (10.6%) out of 3059 ticks, with 5.9% testing positive for *R. aeschlimannii*, marking the first detection of this pathogen in Spain [100]. A study on Iberian red deer in the same region found that 558 of 582 ticks (96%) were adult *H. marginatum*, with 39% testing positive for *A. marginale* [107]. From 2001 to 2005, in La Rioja, 34.3% of 496 ticks collected were identified as *H. marginatum*, with a low prevalence (1.8%) of SFGR [106]. Later, Fernández-Soto et al. [102] identified *H. marginatum marginatum* in 426 out of 4049 ticks collected from humans in Castilla y León between 1997 and 2003, with 6.1% testing positive for *R. aeschlimannii*. Fernández-Soto et al. [101] continued their work, identifying *R. aeschlimannii* in *H. marginatum marginatum* ticks from humans in northwestern Spain, collecting a total of 3853 ticks from 2004 to 2007. Of these, 515 (13.4%) were identified as *H. marginatum marginatum*, with 73 testing positive for *R. aeschlimannii*. Additionally, two male ticks were coinfected with *A. phagocytophilum*, marking the first report of such dual infection in this tick species [101]. Further studies showed *H. marginatum* frequently parasitizing both wildlife and domestic animals. Ruiz-Fons et al. [104] found *H. marginatum* to be the most abundant tick on red deer (63.7%) and wild boar (68.7%). In La Rioja, a study conducted from 2009 to 2011 collected 336 ticks from 19 bird species, with 34 (10%) identified as *H. marginatum* [105]. Palomar et al. [23] analyzed *Hyalomma* ticks from humans and birds in northern Spain between 2009 and 2015, identifying all 161 ticks as *H. marginatum*. Although no ticks tested positive for CCHFV, *R. aeschlimannii* was found in 53 ticks (24.9%) and *R. sibirica* subsp. *mongolitimonae* was found in two ticks (0.9%). Tick surveillance conducted in Castilla y León from 2014 to 2019 collected 734 *H. marginatum* ticks from humans, including two nymphs, 233 females, and 499 males [113].

*In Central Spain*, a survey on tick-borne bacteria found *R. aeschlimannii* DNA in eight of 13 *H. marginatum* ticks (61.5%) out of a total of 1480 adult ticks [111].

*In Eastern Spain*, on Menorca Island, a survey conducted between 1999 and 2000 found that 26.8% (972 out of 3624) of ticks collected from dairy cattle were adult *H. marginatum* [108]. In 2010, a study on Minorca Island tested 14 *H. marginatum* ticks from cattle farms, with one positive for *B. occultans* (14.3%) and two positives for *T. buffeli* (28.6%) [110]. Between 2013 and 2015, 2053 ticks were collected from livestock and vegetation, with 1333 (64.9%) identified as *H. marginatum*, though none tested positive for CCHFV [103]. In 2017, Mateos-Hernández et al. [112] reported 11 *H. marginatum* ticks from Menorca and Castilla-La Mancha, 10 from cattle and one from a human.

*In Western Spain*, the first detection of CCHFV occurred in 2010, when *H. lusitanicum* ticks from red deer in Cáceres tested positive for the virus [163]. A survey conducted from 2011 to 2015 detected CCHFV in only one *H. marginatum* tick (0.4%) collected from cattle in Cáceres [99]. This positive finding was among a total of 238 *H. marginatum* ticks, which comprised 15.1% of the total sample, including 206 ticks from vegetation, 27 from red deer, and five from cattle. Data from Spain reveal a broad and long-standing presence of *H. marginatum*, with detections across northern, central, eastern, and western regions. The species has been recorded on a wide range of hosts and frequently carries pathogens. The involvement of both immature and adult ticks in pathogen transmission, along with evidence of coinfections and regional differences in prevalence, highlights the complex eco-epidemiology of *H. marginatum* in Spain and highlights the importance of continued, regionally tailored surveillance efforts.

Overall, Southern Europe remains the most important ecological and epidemiological hotspot for *H. marginatum* in Europe, with established populations, high host diversity, and frequent pathogen circulation highlighting its central role in tick-borne disease dynamics. Together, records from Italy, Malta, Portugal, and Spain confirm that *H. marginatum* is firmly established in the region, where warm Mediterranean climates support its full life cycle and reproduction. The species is consistently found on a wide range of hosts—including livestock, wildlife, and birds—and across varied habitats, demonstrating its strong adaptability. Importantly, migratory birds play a dual role: not only do they support local tick populations by hosting immature stages, but they also facilitate the species' northward dispersal. This bird-mediated spread introduces *H. marginatum* into new areas, potentially expanding its geographic range under changing environmental conditions.

### 4.2. *Hyalomma marginatum* Distribution in the Balkan Peninsula

The Balkan Peninsula ranks as the second most extensively studied region, after Southern Europe, in research on *H. marginatum*, with 28 studies documenting its presence, accounting for 23% of all publications reviewed. In addition, four studies have reported broader findings, including the presence of *H. marginatum* s.l., *H. marginatum* sensu stricto, the *H. marginatum* complex, and *Hyalomma* species across the region.

The Balkan Peninsula is particularly vulnerable to extensive colonization by *H. marginatum*. Since 1952, the region has experienced both ongoing outbreaks and sporadic cases of CCHF, with *H. marginatum* as the primary vector. However, the tick species *Rhipicephalus bursa* is also recognized as a significant vector for CCHFV after *H. marginatum*. Alongside CCHFV, various species of *Rickettsia* have been identified in *H. marginatum* ticks in this region, suggesting a potential for multiple pathogen transmission.

#### 4.2.1. Albania

The presence of *H. marginatum* has been confirmed through recent surveillance efforts. In a study by Papa et al. [48], 341 *H. marginatum* ticks were collected from livestock, of which 4.7% tested positive for CCHFV. This finding is consistent with Albania's long-standing status as a CCHF-endemic country. The first human case was recorded in 1986 and the northeastern region bordering Kosovo continues to be considered a high-risk area for virus circulation [164]. These data, though limited, point to active enzootic transmission cycles involving *H. marginatum* and its involvement in CCHFV transmission in high-endemic areas.

#### 4.2.2. Bosnia

*Hyalomma marginatum* was first documented in 2008, representing 5.7% of the 10,050 ticks sampled nationwide, with specimens primarily collected from sheep (54.4%), cattle (22.2%), dogs (21.5%), and goats (1.9%) [47]. In a follow-up survey, CCHFV RNA was detected in one pool of three male *H. marginatum* ticks. Another survey later reported that 2.2% of the 6067 ticks collected were identified as *H. marginatum*, primarily found on cattle and sheep [66]. The most recent survey in Bosnia detected CCHFV RNA in one pool of three male *H. marginatum* ticks among a sample of 760 ticks [68]. Although limited in scope, studies in Bosnia consistently report the presence of *H. marginatum* in livestock, with occasional detection of CCHFV RNA. These findings suggest an established population capable of pathogen transmission, warranting expanded surveillance in both livestock and environmental reservoirs.

#### 4.2.3. Bulgaria

The first recorded presence of *H. marginatum* was reported in Carevo in 2006 [40]. Although the exact distribution of *H. marginatum* remains uncertain, Bulgaria has reported multiple cases of CCHF since its initial identification in 1952, with 1105 cases documented between 1953 and 1974 and a fatality rate of approximately 17% [55]. Between 1975 and 2010, 450 additional cases were reported, with a reduced fatality rate of 5.48%, likely due to the introduction of a Bulgarian-developed CCHFV vaccine in 1974 [55]. Bulgaria is now considered endemic for CCHFV, posing an ongoing public health risk. From 2006 to 2010, *H. marginatum* was the most prevalent tick species in endemic regions, accounting for 31.2% of 911 adult ticks collected from livestock, and 14 of 284 *H. marginatum* specimens tested positive for CCHFV RNA [55]. In 2014, a survey across five districts found that 60.5% of 1030 *H. marginatum* ticks tested positive for CCHFV RNA [151]. Recent research has also identified *H. marginatum* on Eurasian eagle-owls (*Bubo bubo*) in Southeastern Bulgaria, with 15 ticks collected from this host, further confirming the species' widespread presence in the country [62]. Bulgaria is one of the most extensively studied Balkan countries for both *H. marginatum* and CCHFV, with sustained surveillance confirming the tick's central role in virus transmission. Decades of data and control efforts highlight its critical role in the country's endemic transmission cycle.

#### 4.2.4. Croatia

The presence of *H. marginatum* is well-established along the Mediterranean coast of Europe and is documented in Croatia. In a tick survey conducted in 2000 in Split, Dalmatia County, 17 out of 197 collected ticks (8.6%) were identified as *H. marginatum*, with 64.7% of these specimens (11 individuals) testing positive for *R. aeschlimannii* [61]. Despite limited understanding of the epidemiology of rickettsial diseases in Croatia, two tick-borne rickettsial illnesses have been reported in the southern part of the country: Mediterranean spotted fever, caused by *R. conorii*, in coastal regions and rickettsial pox, caused by *R. akari*, in northern areas [61]. A separate study investigating rickettsiae in Croatian ticks found *R. conorii* present in 12.6% of *R. bursa* ticks, 10.6% of *R. sanguineus* ticks, and 7.3% of *Dermacentor marginatus* ticks; however, *H. marginatum* was not included in that survey [165]. The most recent data, covering tick collections from 2017 to 2020, documented three *H. marginatum* specimens collected from vegetation [67]. Data from Croatia indicate a scattered presence of *H. marginatum*, mainly in coastal areas. Despite sparse records, the detection of *R. aeschlimannii* in local specimens signals a possible, though underreported, contribution to the region's tick-borne pathogen landscape.

#### 4.2.5. Greece

*Hyalomma marginatum* and various other ixodid tick species have been identified in domestic animals and humans, with numerous studies investigating *H. marginatum* distribution and associated pathogens. The first and largest tick survey on domestic animals was conducted in Macedonia between 1983 and 1986, with 11,620 ticks collected from cattle, sheep, goats, and dogs. *Hyalomma marginatum marginatum* comprised 3.5% of the total ticks collected, while *R. bursa* was the most abundant species, comprising 36.3% of the collection. Additionally, adult *H. marginatum rufipes* and *H. marginatum turanicum* were observed on cattle [65]. A survey conducted in 1998–1999 on Cephalonia Island found 130 *H. marginatum* specimens (7% of the 1848 ticks collected), with two specimens testing positive for *C. burnetii*, though no ticks tested positive for rickettsia [51].

In a survey conducted from 2003 to 2006 in Northern Greece, a total of 3249 adult ticks were collected from goats and sheep, with 12.4% identified as *H. marginatum* [50]. This species was also commonly found in migratory birds, as shown by surveys from 2009 to 2010 at bird observatories in Capri, Italy, and Antikythira, Greece, where 88.2% (659 of 747) of ticks collected were *H. marginatum* [166]. In subsequent testing of 13,332 migratory birds, *H. marginatum* s.l. accounted for 90% of the ticks collected. Pathogen screening of *H. marginatum* s.l. ticks revealed positive results for *R. aeschlimannii* (45.6%), *Rickettsia* spp. (2.4%), and *R. africae* (2.3%) [24]. Additionally, one *H. marginatum* sensu stricto specimen was identified that tested positive for *R. aeschlimannii*.

Recent surveys indicate a decline in *H. marginatum* presence, with only 0.2% of ticks collected from livestock identified as *H. marginatum* between 2012 and 2013 [53]. Although CCHFV assessments in Greece reported no human cases until 2008, 2.8% of tick pools collected between 2012 and 2014 tested positive, predominantly in *R. sanguineus* and *R. bursa* samples, while all *H. marginatum* samples tested negative [167].

*Hyalomma marginatum* is widely distributed across Greece and has been detected on domestic hosts, migratory birds, and humans. Although recent studies show a decline in prevalence, the tick's frequent association with *Rickettsia* species and past CCHFV surveillance affirm its continued epidemiological relevance.

#### 4.2.6. Kosovo

Tick collections conducted in between 2001 and 2014 confirmed *H. marginatum* presence across Kosovo [54, 58, 59]. The country experiences periodic CCHF outbreaks, with the first major outbreak documented in 1970 among shepherds near the border with North Macedonia [17]. However, the earliest known case dates back to 1954. Since 1989, Kosovo has experienced regular CCHF outbreaks approximately every 4–5 years [168, 169]. Following the initial 1970 outbreak, another significant outbreak occurred between 1991 and 1992, with subsequent outbreaks in 1995 (65 patients, seven fatalities) and during the period of 1996–2000 (33 sporadic cases, seven fatalities). In 2001, shortly after the Kosovo war, the largest epidemic was reported with 155 suspected and 30 confirmed cases [17]. According to Jameson et al. [170], around 50% of Kosovo's municipalities are considered at risk for CCHF, with highly endemic areas identified in Skënderaj, Klinë, Malishevë, Rahovec, and Suharekë. There are two distinct genetic lineages of CCHFV in Kosovo and Albania. The Europe 1 (Clade V) lineage, primarily vectored by *H. marginatum*, is highly pathogenic to humans. In contrast, the Europe 2 (Clade VI) lineage, vectored by *R. bursa*, exhibits mild or nonpathogenic characteristics in humans [48]. The frequent circulation of CCHFV in ticks, combined with repeated human outbreaks in southwestern Kosovo and northern Albania—where *H. marginatum* is prevalent—strongly suggests the presence of the highly pathogenic Europe 1 strain in these regions [54].

Kosovo remains a high-risk area for CCHFV, with *H. marginatum* playing a central role in the transmission of the Europe 1 strain. Recurring outbreaks and consistent tick positivity indicate the tick's well-established presence and epidemiological importance in endemic zones.

#### 4.2.7. Romania

The first report of *H. marginatu*m dates back over 55 years, as documented in Feider's review of tick distribution [44]. While *I. ricinus*, *D. marginatus*, and *Haemaphysalis punctata* are more prevalent nationwide, *H. marginatum* is primarily found in Southern Romania. Between 1998 and 2004, a livestock surveillance study identified only 6.9% of 2706 ticks as *H. marginatum* [64]. Subsequent studies have confirmed this species' presence on vegetation, mammals, and birds. For example, a survey reported just 0.02% (2 out of 13,771) of ticks collected from vegetation as *H. marginatum* and 2.4% (20 out of 840) from animals [44, 57]. In 2010, 35.3% of 382 ticks collected in a study on tick-borne pathogens were identified as *H. marginatum*, with *Babesia* spp. and *Theileria* spp. DNA detected in 12 (8.9%) of these ticks, showing sequences similar to *T. equi*, *T. orientalis/sergenti/buffeli*-group, and *B. occultans* [52]. In 2013, three adult *H. marginatum* ticks were reported from dogs in Southern Romania [56]. Additional findings in the Danube Delta Biosphere Reserve between 2012 and 2013 detected 32 ticks on birds, 31 of which were *H. marginatum marginatum*, with one specimen collected from a juvenile song thrush testing positive for WNV [45]. A study on urban crows in 2013 also identified one *H. marginatum marginatum* nymph from a Eurasian jackdaw (*Corvus monedula*) [49]. In Romania, *H. marginatum* appears to be locally established, particularly in southern regions. Although less abundant than other tick species, it has been found to harbor several pathogens, including *Babesia*, *Theileria*, and WNV, highlighting its medical and veterinary relevance.

#### 4.2.8. Other Balkan Countries

Reports also confirm the presence of *H. marginatum* in North Macedonia, Montenegro, and Serbia, although specific data on its distribution and pathogen prevalence in these countries remain limited [22].

The Balkan Peninsula is a critical region for understanding *H. marginatum* and its role in transmitting zoonotic pathogens like CCHFV and *Rickettsia* spp. While significant progress has been made in mapping its distribution and pathogen associations, some countries in the region remain underrepresented in the data, leaving critical gaps in knowledge. Targeted studies and improved diagnostics are essential for developing effective control strategies to mitigate the impact of *Hyalomma*-borne diseases in the region.

### 4.3. *Hyalomma marginatum* Distribution in Western Europe

A total of 18 publications were included from Western Europe, accounting for 14.1% of all the studies analyzed.

#### 4.3.1. France

*Hyalomma marginatum* is widely distributed in the southern regions of the country, where the climate, high density of migratory birds, and the presence of ungulates, especially horses, create favorable conditions for its establishment.

*In Southern France*, between 2007 and 2010, a survey identified five *H. marginatum* ticks (1.2%) out of 406 collected, with one tick infected with *A. phagocytophilum* [73]. While historical records have sometimes been inconsistent, recent studies have confirmed the established presence of *H. marginatum* in Southern France. Comprehensive tick collections conducted from 2007 to 2016 on horses and birds identified 84 *H. marginatum* ticks (7.1%) out of a total of 1179 specimens [71].

In the Camargue region, a study conducted between 2015 and 2016 identified 47 *H. marginatum* out of 585 horse ticks, with 43% positive for *T. equi*, a significant pathogen in equine piroplasmosis [78]. *Hyalomma marginatum* was less common in wet habitats of Camargue, aligning with its xerophilic nature, which favors drier environments like Corsica. These findings reinforce the expanding range of *H. marginatum* in France and its critical role in transmitting pathogens such as *Rickettsia*, *Theileria*, and *Ehrlichia* across diverse hosts. From 2016 to 2019, a survey of ticks collected from horses across 14 French departments in the Mediterranean corridor of Southern France found that *H. marginatum* comprised 36% of the 2588 ticks collected [81].

*In Corsica*, a favorable climate and extensive livestock farming have allowed *H. marginatum* to thrive. Initial records date back to 1959, with additional reports in 2004 and 2007 [76]. This survey collected 3134 ticks, of which 571 (18.2%) were *H. marginatum*, predominantly on cattle (73.2%) and horses (24.5%). Pathogen analysis detected *R. aeschlimannii*, *Francisella*-like endosymbionts, *A. marginale*, and *A. phagocytophilum* in these ticks [29]. Since 2015, large-scale tick surveys in Corsica have significantly enhanced understanding of *H. marginatum* distribution and its role in pathogen transmission. In 2015, Dahmani et al. [75] identified five *H. marginatum* (4.1%) out of 123 ticks collected. In 2016, 91 *H. marginatum* ticks (13.8%) were found among 660 collected from cattle, making it the second most abundant species after *R. bursa* [70]. Molecular analysis detected *Rickettsia* spp., *B. burgdorferi* (s.l.), *Anaplasma* spp., and *E. minasensis* DNA, marking the first identification of *E. minasensis* in *H. marginatum* in Corsica [70, 74]. From 2017 to 2019, surveys collected 216 *H. marginatum* ticks (19.3%) from cattle and wild boar out of 1117 total ticks, all of which tested positive for *R. aeschlimannii* [28]. Concurrently, in Palasca and Nessa, 216 *H. marginatum* ticks (9.8%) out of 820 samples tested positive for *A. phagocytophilum* and *E. minasensis* DNA, highlighting the species' widespread presence and its involvement in pathogen transmission [72]. Recent studies across Corsica and France highlight the abundance of *H. marginatum* in livestock and wild animals. Between 2018 and 2019, 3555 ticks were collected from sheep, cattle, wild boars, and horses in Corsica, with 1454 identified as *H. marginatum* [77]. Parapoxvirus DNA was detected in 8.2% of *H. marginatum* pools. In a separate study (2018–2020), 113 ticks were collected from wild boars, with two identified as *H. marginatum*, and one (50%) positive for *R. aeschlimannii* [79]. From 2019 to 2021, 702 *H. marginatum* ticks from Corsican cattle tested positive for *R. aeschlimannii*, with 4.3% also positive for *Theileria* spp. and *E. minasensis* [80].

France—especially its southern regions and Corsica—has seen a marked increase in *H. marginatum* detections across multiple ecosystems. The tick's adaptation to drier climates, strong presence on livestock and equids, and consistent detection of multiple pathogens in both mainland and island contexts suggest an expanding ecological niche and a rising risk of tick-borne disease transmission in Mediterranean France.

#### 4.3.2. Netherlands

The first documented exotic *Hyalomma* species was *H. aegyptium*, reported by Bronswijk et al. in 1979 [171]. Between 2012 and 2014, tick collections on birds in the Netherlands confirmed the presence of imported *Hyalomma* species in the country [172]. While immature stages have been sporadically found on migratory birds, adult stages have been reported less frequently in the Netherlands. According to Uiterwijk et al. [39], three adult *Hyalomma* ticks were identified on horses between 2005 and 2009, one of which was identified *as H. marginatum rufipes*. No pathogens were detected in these specimens [171]. Exotic tick species, such as *Hyalomma*, are occasionally introduced into the Netherlands via imported reptiles, pets, migratory birds, or companion animals returning from endemic areas [39]. From 2019 to 2020, reports of *Hyalomma* ticks were documented through a citizen science project [39]. One adult *Hyalomma* species was found in 2018 and reported in 2020, with 17 additional specimens identified as adult *Hyalomma* species ticks (one in 2018, 11 in 2019, and five in 2020). Based on morphological and molecular species identification, along with cluster analysis, 12 of these ticks were confirmed as adult *H. marginatum*. Nearly all of the specimens (11 out of 12) were discovered on horses, with one adult tick collected from a human. Pathogen detection analysis revealed that one adult female *H. marginatum*, reported from a horse in 2019, tested positive for *R. aeschlimannii*.

*Hyalomma marginatum* is not considered established in the Netherlands, but is occasionally detected, predominantly through introductions via migratory birds and horses. Although these records are rare and mostly involve adult ticks, the occasional detection of pathogens such as *R. aeschlimannii* warrants ongoing surveillance. The role of citizen science has proven valuable in documenting these sporadic occurrences, reinforcing the importance of public engagement in early warning systems, particularly as climate change and animal movement patterns evolve.

#### 4.3.3. United Kingdom

Sporadic *H. marginatum* records date back to historical data compiled by the Biological Records Centre (1860–2001) [42]. Between 2005 and 2009, one *H. marginatum* specimen was found among 4172 ticks, marking the first adult record in the United Kingdom, likely transported from Portugal [42]. In 2010 and 2011, 14 nymphal *H. marginatum* ticks (21%) were collected from bird species such as sedge warbler, northern wheatear (*Oenanthe oenanthe*), common redstart, and whitethroat, though none tested positive for CCHFV [29]. Over the past three decades, *H. marginatum* has been intermittently introduced to the United Kingdom through passive transport on migratory birds flying northward, yet no permanent populations of this species have been established. This is likely due to the unsuitable climatic conditions in the United Kingdom, where the summers are typically too wet or too cold for the tick's full development [173, 174]. A recent 2021 report documented the first human exposure to an adult *H. marginatum* in England, identified as *H. marginatum* and positive for *R. aeschlimannii* [82]. Additionally, a 2018 finding in England involved a male *H. rufipes* on a horse with no travel history, also testing positive for *R. aeschlimannii*, potentially indicating a successful molt within the United Kingdom [156].

Despite repeated introductions of *H. marginatum* into the United Kingdom, primarily through migratory birds and occasional detections on horses and humans, environmental conditions currently remain unsuitable for stable establishment. The findings of pathogen-positive specimens emphasize potential health risks, reinforcing the importance of continuous surveillance amid changing climate conditions and animal movement patterns.

Western Europe reflects a gradient—from established foci in the south to passive introduction zones in the north—highlighting the need for region-specific monitoring strategies. In this region, *H. marginatum* is well-established in Southern France and Corsica, but remains sporadically introduced into the United Kingdom and the Netherlands. Its repeated detection alongside pathogens highlights potential health risks, necessitating continued surveillance under changing climate conditions.

### 4.4. *Hyalomma marginatum* Distribution in Central Europe

A total of 13 studies in Central Europe (10.7% of all publications) report the presence of *H. marginatum* across various countries in this region; however, no permanent populations have been established.

#### 4.4.1. Austria

The first recorded occurrence of *H. marginatum* was in 2018, when a male tick was found on a horse in Lower Austria [31]. Likely introduced by migratory birds, the tick tested negative for CCHFV, but positive for *R. aeschlimannii*.

The detection of *H. marginatum* on domestic animals highlights the potential for tick introductions linked to migratory birds and animal transport. Ongoing surveillance and pathogen monitoring are, therefore, essential to detect potential establishment and associated disease risks.

#### 4.4.2. Czechia

According to Capek et al. [84], immature stages of *H. marginatum* complex ticks were detected on migratory birds in the former Czechoslovakia over 60 years ago. Three nymphs were collected from tree pipits (*Anthus trivialis*) and one nymph from a bluethroat (*Luscinia svecica*) in 1953 [175]. Surveys from 2010 to 2012 also identified larval and nymphal stages of the *H. marginatum* complex on migratory birds in Central Moravia and Eastern Bohemia. Specifically, 12 ticks were collected in 2010 from great reed warblers and common nightingales (*Luscinia megarhynchos*); in 2011, one adult female was found on an Eurasian reedv warbler; and in 2012, two ticks were collected from marsh warblers (*Acrocephalus palustris*) and common nightingales [84]. These host species are long-distance migratory birds that winter in sub-Saharan Africa and are likely responsible for introducing *H. marginatum* into Central Europe during their spring migration from North Africa or the Mediterranean basin.

*Hyalomma rufipes* has also been documented in Czechia. In 2019, a male *H. rufipes* tick was collected from a horse in South Moravia [175], followed by another adult male specimen in April 2020 from the same location [176]. Testing of these specimens for flaviviruses, bunyaviruses, phleboviruses, and *Rickettsia* spp. yielded negative results. Recent studies also documented four adult *H. marginatum* ticks on horses and one from a household, totaling nine *Hyalomma* ticks, including one nymph on a ringed common nightingale [32]. Molecular analysis confirmed both *H. marginatum* and *H. rufipes*, marking the first molecular identification of these species in Czechia. Recent detections of immature *H. marginatum* and *H. rufipes* ticks on migratory birds in Czechia highlight the country's role as an important transit area for these species into Central Europe. Although consistent findings indicate repeated seasonal introductions, there remains no evidence to support the establishment of stable local populations.

#### 4.4.3. Germany

Although *H. marginatum* is not part of the indigenous tick fauna in Germany, its presence has been sporadically reported in recent years. The first documented case occurred in 2006, when a questing female *H. marginatum marginatum* was found on a person's clothing after spending time in rural areas. Molecular testing for CCHFV and *R. aeschlimannii* yielded negative results [83]. Migratory birds regularly transport immature stages of *H. marginatum* into Germany during spring migration. In a study by Rumer et al. [35], 294 ticks were collected from birds in the Berlin–Brandenburg region; molecular analysis identified three as *H. marginatum* complex, all positive for *R. aeschlimannii*. Another occurrence was reported in 2016 when a female *H. marginatum* was found on a man's trousers in Tübingen, marking the first confirmed morphological and genetic identification of the species in Germany [34]. In a 2018 survey by Chitimia-Dobler et al. [36], 10 out of 18 ticks collected across various districts were identified as *H. marginatum*, with 50% testing positive for *R. aeschlimannii*. The remaining eight ticks were *H. rufipes*; three collected from horses and one from a horse's habitat tested positive for *R. aeschlimannii*. Additionally, in December 2015, *H. rufipes* was first reported in Germany on a horse in Rhineland-Palatinate, with negative tests for rickettsiae and CCHFV [177]. Germany's repeated detection of *H. marginatum*, particularly linked to migratory birds and sporadic human encounters, highlights its ongoing but transient presence. The country's climatic conditions currently limit permanent establishment, yet the potential for pathogen introductions necessitates sustained surveillance efforts.

#### 4.4.4. Hungary

Sporadic records of *H. marginatum* have been noted from imported animals, humans, and migratory birds. In 2009, Földvári et al. [85] identified a nymphal *H. marginatum* feeding on a hedgehog in a Budapest park. A 2011 survey on cattle and wild ruminants found two adult *H. marginatum rufipes* males on cattle, marking the first record of this species on cattle in Central Europe [178]. This area was previously identified by Estrada-Peña et al. [173] and Celina et al. [179] as high risk for *Hyalomma* establishment. The first molecular evidence of *H. marginatum* in Hungary came from Hornok et al. [37], who identified three immature ticks on a robin (*Erithacus rubecula*), with two testing positive for *R. aeschlimannii* (66.6%). These findings, combined with seropositivity rates among animals, suggest Hungary could be a new region for CCHFV distribution [180]. Magyar et al. [180] found CCHFV exposure in 12 out of 2700 human serum samples, although the limited presence of *H. marginatum* does not indicate stable populations in the country.

In 2021, a citizen-science project recorded two *Hyalomma* specimens: a male *H. marginatum* found in a dog and a male *H. rufipes* feeding on a cow [38]. These findings highlight the sporadic nature of *Hyalomma* ticks in Hungary and the need for ongoing surveillance. Between 2012 and 2014, a total of 3339 ixodid ticks were collected from 1167 passerine birds in Hungary, including three *H. rufipes* nymphs on a common whitethroat in May 2014, marking the first molecular evidence of avian transport of immature *H. rufipes* in Central Europe [181]. In 2022, 956 ixodid ticks were collected from birds in Hungary, including 12 *H. rufipes*, were found on various species: one nymph on a common whitethroat, one on a European pied flycatcher (*Ficedula hypoleuca*), three on sedge warblers, and eight on bearded reedlings (*Panurus biarmicus*), comprising five nymphs and one larva [182].

Hungary's intermittent findings of *Hyalomma* ticks across diverse host species, alongside evidence of pathogen presence and human CCHFV exposure, reflect periodic introductions and underline the need for proactive tick and pathogen surveillance.

#### 4.4.5. Poland

Historical records indicate the presence of *H. marginatum* in the 1930s and 1940s, with four unfed male specimens found in Bytom, Upper Silesia, preserved in the Bytom Museum [40]. More recent records are limited, with only two findings on migratory birds: one on a western yellow wagtail and another on a sedge warbler [183]. A recent epidemiological study of CCHFV in cattle in southeastern Poland did not detect *H. marginatum*, identifying only *D. reticulatus* and *I. ricinus* instead [184]. Current evidence of *H. marginatum* in Poland is scarce, limited to occasional detections, suggesting transient occurrences rather than established populations. Continued monitoring remains necessary to clarify its status.

#### 4.4.6. Slovakia

*Hyalomma marginatum* has also been recorded on migratory birds. Historical records show a nymph of *H. marginatum* complex collected from a robin in 1953 and two larvae and four nymphs from a marsh warbler in 1987 [175]. Additionally, in 1981, a unique instance documented a female *H. marginatum* on a man's clothing [175]. More recently, between 2008 and 2009, immature stages of the *H. marginatum* complex were found on migratory birds in southwestern Slovakia, including species similar to those in Czechia, such as the great reed warbler, Eurasian reed warbler, sedge warbler, and Savi's warbler (*Locustella luscinioides*) [84].

Immature *Hyalomma* ticks have been sporadically detected on passerines in Slovakia; however, species-level confirmation is limited. Further research is needed to determine whether *H. marginatum* specifically occurs and whether conditions could support its establishment.

Overall, In Central Europe, *H. marginatum* is not yet established. Its presence is sporadic and primarily linked to passive introduction of immature stages via migratory birds during spring. These ticks rarely develop into adults and there is no evidence of local reproduction or sustained life cycle completion. Most records involve larvae and nymphs on birds, while occasional adult ticks on livestock or humans likely reflect recent importation or local molting events, rather than true colonization. Importantly, despite repeated introductions, climatic conditions in the region are likely insufficient to support full tick development and overwintering. Despite this, some introduced ticks carry pathogens like *R. aeschlimannii*, posing a risk of pathogen introduction. As climate change alters environmental suitability, Central Europe may become increasingly vulnerable to colonization. The patterns observed emphasize the region's role as a sentinel front for tracking early signals of northward tick expansion under changing climatic and ecological pressures.

### 4.5. *Hyalomma marginatum* Distribution in Eastern Europe

Eastern Europe, with the fewest recorded publications on *H. marginatum* (only 4.7% of the total), likely harbors a significant population, especially in Southern Ukraine and Russia. However, ongoing conflict in these areas has severely limited research capabilities, making it nearly impossible to study the actual abundance and distribution of *H. marginatum*. War-driven changes in land use, such as abandoned farms, displaced livestock, and altered human activities, may also increase the risk of CCHF outbreaks by creating favorable conditions for tick proliferation and pathogen transmission.

#### 4.5.1. Moldova

*Hyalomma marginatum* had not been reported in the country since the 1980s. However, systematic surveillance during the spring seasons of 2012 and 2015 identified four specimens on migratory birds, confirming its reappearance in the country [87]. Ongoing monitoring is essential to assess whether these introductions could lead to more consistent seasonal presence.

#### 4.5.2. Russia

*Hyalomma marginatum* is extensively distributed in Southern Russia, where it poses a major public health concern. This tick species is prevalent in several Russian regions, including Stavropol, Rostov, Krasnodar, Dagestan, and the Karachay-Cherkess Republic, all recognized as CCHF-endemic areas where *H. marginatum* plays a dominant role in the virus's transmission [153]. The tick was associated with periodic CCHF outbreaks in Russia, notably resurging in 1999, when 40 CCHF cases were reported [152]. In European Russia, sporadic cases of the CCHF are registered each year. During 2000 and 2001, 133 primary cases of CCHF were identified in European Russia, with a fatality rate of 9.0% [185]. In Southern Russia, *H. marginatum* accounts for nearly 50% of ticks collected from domestic animals, with studies confirming the presence of pathogens in this species. For instance, Yashina et al. [152] examined 4787 *H. marginatum* ticks from European Russia, detecting CCHFV in 10.2% of 449 pools. Similarly, Tsapko et al. [153] found *H. marginatum* to be the dominant species among over 38,000 ticks, comprising 66% of the specimens, with 81% of the CCHFV-positive pools from this species. In May 2004, Shpynov et al. collected 40 adult *H. marginatum* ticks (20 females and 20 males) from cattle and vegetation in Stavropol, with 20% testing positive for *R. aeschlimannii*. *Hyalomma marginatum* has also been documented outside Southern Russia. Movila et al. [89] recorded five *H. marginatum* on migratory birds in Kaliningrad Oblast, marking the first detection in Russia's Baltic region. In one nymph collected from a tree pipit, *R. aeschlimannii* was detected. The Stavropol and Rostov regions remain notable hotspots for CCHFV, largely due to the high density of *H. marginatum* populations.

*Hyalomma marginatum* is firmly established in Southern Russia and remains a dominant vector of CCHFV in endemic regions. Its high abundance, coupled with consistent pathogen detection, highlights its central role in the country's tick-borne disease ecology. Sporadic findings beyond its core range point to a potential for further expansion.

#### 4.5.3. Ukraine

The distribution of *H. marginatum* in Ukraine extends along Northern Odesa, Mykolayiv, Kirovograd, Dnipropetrovsk, Zaporizhia, Donetsk, Luhansk Oblasts, and the Crimean peninsula, with widespread presence in the southern regions. Further, regular findings in atypical locations suggest potential for spreading zoonotic diseases to new areas, increasing epidemic risks [90]. The first reports of *H. marginatum* in former Soviet union date back to the 1940s, in the context of CCHFV outbreaks in the Crimean region.

From 1977 to 2009, researchers conducted comprehensive surveys across Crimea, 22 other Oblasts, and major cities, including Kyiv, Mariupol, and Sevastopol, to determine the range of *H. marginatum* in Ukraine [90]. Approximately 12,000 ticks were collected using flag-dragging and host examination methods, with 78% of adult *H. marginatum* collected from vegetation and 22% from animal hosts.

In Ukraine, *H. marginatum* is well-established across southern parts of the country. Historical associations with CCHFV highlight the country's relevance in the regional ecology of tick-borne diseases. Recent military conflicts and large-scale population displacements may disrupt local ecosystems, alter host movements, and reduce surveillance capacities, collectively increasing the risk of tick expansion and zoonotic pathogen transmission.

Eastern Europe, particularly Southern Russia and Ukraine, harbors long-established populations of *H. marginatum*, playing a central role in CCHFV transmission. Historical outbreak data and high tick abundance confirm its vector importance in these endemic zones. Despite low publication numbers, surveillance shows the species is well adapted to dry steppe and agricultural habitats. However, ongoing conflicts and limited research capacity have hampered recent monitoring efforts. This region combines a strong ecological foundation for tick-borne diseases with an urgent need for renewed surveillance and control programs.

### 4.6. *Hyalomma marginatum* Distribution in Northern Europe

The recorded publications on *H. marginatum* in Northern Europe are limited, accounting for only 3.9% of the total analyzed studies. All reported occurrences of *H. marginatum* in this region have involved immature stages, which were likely transported via migratory birds during their spring migration northward.

#### 4.6.1. Denmark

The first recorded instance of *H. marginatum marginatum* in Northern Europe dates back to June 1939, when it was found on the island of Bornholm, Denmark, likely carried as a nymph by a migratory bird from the Mediterranean or Africa [186]. Similar evidence of bird-mediated tick dispersal was noted by Jaenson et al. [86], who documented a nymph on a common kestrel in 1991 on the Danish island of Christiansø. *Hylomma marginatum* has been rarely detected in Denmark, with records limited to migratory birds. While establishment is unlikely under current climatic conditions, the country's northern position makes it relevant for monitoring future range shifts.

#### 4.6.2. Finland

Research on ticks parasitizing birds remains limited, with only a few studies documenting tick species detected on avian hosts [187–190]. These studies have reported the presence of four tick species: *I. ricinus*, *I. arboricola*, *I. lividus*, and *H. marginatum*. A historical record noted *H. marginatum* on a migratory bird [189] and a recent survey (2018–2020) found *H. marginatum* in 0.7% of 434 ticks collected from 32 bird species [33]. Specifically, two specimens were found on a barred warbler (*Curruca nisoria*) and one was collected on a tree warbler (*Iduna caligata*). Although research is limited, both historical and recent studies confirm low-frequency introductions of *H. marginatum* via migratory birds. The detection of the species in recent bird surveillance highlights the need for continued avian-based monitoring.

#### 4.6.3. Norway

Similarly, *H. marginatum* has occasionally been detected on migratory birds in Norway. The first records of *H. marginatum* nymphs were reported from common redstart, Eurasian reed warbler, willow warbler, and red-backed shrike (*Lanius collurio*) [86]. Since these early reports, no further scientific studies have confirmed the presence of *H. marginatum* in Norway, though occasional media reports have mentioned sightings. In addition to these records, a study conducted between 2003 and 2005 identified seven fully engorged nymphs of *H. rufipes* on six bird species, including the garden warbler (*Sylvia borin*), common redstart, reed warbler, wheatear, whitethroat, and two individuals of the thrush nightingale (*Luscinia luscinia*)—all of which are bird species that winter in Africa [191]. The absence of confirmed adult specimens and follow-up detections suggests minimal risk of establishment in Norway, though occasional monitoring remains warranted.

#### 4.6.4. Sweden

The initial reports of *H. marginatum* nymphs on birds date back to the early 1990s [86]. However, a more recent survey between 2018 and 2019 recorded over 40 adult *Hyalomma* ticks found on horses, cattle, and humans across various provinces [26]. Among these, 11 specimens were morphologically identified as *H. marginatum*, marking the first documented presence of adult *H. marginatum* in Sweden. Although testing for CCHFV and piroplasms was negative, four specimens tested positive for *R. aeschlimannii*, indicating potential zoonotic risks [26].

In Northern Europe, *H. marginatum* occurrences remain sporadic and largely limited to immature stages introduced by migratory birds. The climatic conditions currently inhibit molting, development, and overwintering, thereby preventing local establishment. However, rare reports of molted adult ticks, particularly in Sweden, suggest that under increasingly favorable microclimatic conditions, individual ticks may complete development after arrival. While the risk of establishment remains low, ongoing climate change and repeated introductions across multiple countries highlight the need for continued ornithological and passive surveillance to detect early ecological shifts and emerging tick-borne disease risks.

## 5. Overview of Regional Findings and Study Limitations

The findings from this review reveal that *H. marginatum* distribution is concentrated mainly in the Mediterranean and Balkan regions of Europe, with sporadic occurrences in Western, Central, Eastern, and Northern Europe. This distribution is consistent with studies showing that *H. marginatum* is well-suited to warm and arid climates characteristic of Mediterranean and sub-Mediterranean areas, where the tick has traditionally established stable populations [14, 173]. Additionally, evidence of *H. marginatum* dispersal into more temperate regions through migratory birds supports the notion that these birds contribute to the seasonal transport of tick species, facilitating their introduction into nonendemic areas [192, 193]. However, the environmental constraints—particularly cooler temperatures and higher humidity—of Central and Northern Europe have so far hindered the establishment of stable *H. marginatum* populations in these regions. This pattern is also observed in studies of other tick species, which tend to expand their ranges in response to climate change, indicating that projected warming could make northern habitats more favorable for *H. marginatum* colonization in the future [194, 195].

Beyond climatic constraints, geopolitical instability further complicates tick surveillance efforts, particularly in regions experiencing military conflicts. Armed conflicts disrupt health infrastructure, impede vector surveillance programs, and create ecological conditions that favor tick population growth and disease emergence. Despite the significant role that war-related environmental disturbances play in the epidemiology of tick-borne diseases, this factor remains largely overlooked in existing literature. Historical records indicate that conflict-induced ecological changes have directly contributed to *H. marginatum* population surges and increased human exposure to CCHFV. For instance, during World War II in Crimea, the widespread destruction of farmland and the sharp decline in livestock led to an overpopulation of wild hares—key hosts for immature *H. marginatum*—which in turn resulted in heightened tick exposure among soldiers and agricultural workers, triggering an outbreak of CCHF [7, 196]. A similar situation occurred in Bulgaria in the 1950s, when enforced collectivization of agriculture converted natural landscapes into farmland, increasing human exposure to *Hyalomma* ticks and their small vertebrate hosts, contributing to a surge in CCHF cases [18]. In Turkey, a comparable pattern emerged when residents returned to farmlands that had been abandoned during civil strife, leading to increased human exposure to *H. marginatum* populations sustained by small vertebrate hosts [18]. More recently, conflict-related surveillance gaps have been observed in Iraq, where war and civil unrest halted tick surveillance for nearly a decade, allowing CCHF cases to emerge undetected until recently [197]. Similarly, in Ukraine, a country currently experiencing military conflict, surveillance efforts have been deprioritized due to instability, potentially creating unmonitored transmission hotspots [198]. Environmental disruptions caused by war, including land abandonment and altered land use, can create new habitats favorable for tick proliferation [196]. In regions where small mammals such as hares and hedgehogs thrive while large mammal populations decline, humans become the primary blood source for adult *H. marginatum*, exacerbating disease transmission risks, as observed in historical CCHF outbreaks [18].

A significant limitation of our review is the inconsistency in data collection methods and reporting standards across different studies. Variations in sampling techniques, host selection, and pathogen screening protocols creates difficulties in forming a directly comparable dataset and this inconsistency limits the generalizability of findings. Further complicating the issue is the challenge of accurate taxonomic classification, especially in distinguishing between *H. marginatum* and morphologically similar species like *H. rufipes*. While this review includes studies on *H. rufipes* and other species within the *H. marginatum* complex to account for classification variability, studies that did not provide molecular confirmation of species may have led to potential underrepresentation of the species' distribution. Another limitation relates to the geographic focus of surveillance efforts. Much of the available data focuses on certain high-risk regions (e.g., Southern Europe), which may overlook sporadic occurrences in less-sampled areas. Additionally, tick collection is often concentrated on certain hosts (e.g., livestock or migratory birds), potentially missing data from other wildlife species. Furthermore, we acknowledge that our review may not be entirely comprehensive, as studies published in non-English languages were not included, which could have led to the omission of relevant research findings.

## 6. Future Challenges

*Hyalomma marginatum*, a principal vector of CCHFV in Europe, is expanding its geographic range under the combined pressures of climate change, anthropogenic land use changes, and animal movements. While the introduction of immature stages via migratory birds is well documented [199], the establishment of stable populations requires a confluence of favorable climatic conditions and the availability of suitable vertebrate hosts, particularly large mammals such as cattle, horses, goats, and sheep. Surveillance strategies must, therefore, go beyond sporadic tick collections and include coordinated monitoring of birds, livestock, land use, and environmental variables, particularly in regions currently considered CCHF-free but environmentally suitable for *Hyalomma* species.

One of the key challenges in CCHFV monitoring is the virus's “invisible” circulation in nature. Ticks—especially of the *Hyalomma* genus—are considered not only vectors but also potential reservoirs of CCHFV [200]. However, their vector competence can only be verified through complex and high-cost experiments in biosafety Level-4 laboratories, which are not accessible in many regions. Consequently, the virus may remain undetected until human clinical manifestations appear, masking the broader epidemiological picture. This highlights the need for proactive and multilevel surveillance that includes both vector and vertebrate host populations to identify areas with silent virus presence.

Climate change is a critical driver of *H. marginatum* expansion. Rising temperatures may enhance tick survival and facilitate range extension into northern latitudes previously deemed unsuitable [22]. While warmer conditions support tick development, moderate humidity levels are essential to prevent desiccation during off-host stages. This species typically thrives in semiarid environments characterized by hot and dry summers, but cannot establish in regions that are either too humid or overly arid. Recent studies suggest that *H. marginatum* populations are sustained in areas where summer precipitation remains below approximately 32 mm and relative humidity is around 70% [81]. However, excessively arid conditions with high evaporative stress can limit its colonization, even in regions experiencing temperature increases. Conversely, highly humid environments are also unsuitable. While climate warming opens new territories, only regions with sufficient but not excessive humidity may support permanent populations. Currently, Southern Europe and Northern Africa populations are regulated by summer rainfall, while Eastern Europe and the Caucasus populations are controlled by autumn temperatures [22]. Warmer climates may lower tick mortality rates, aiding their spread into suitable areas [174]. Climate projections suggest that warming trends could reduce tick mortality and enable further northward expansion, potentially surpassing the 47°N latitude boundary [173, 179, 201].

Environmental events, such as the unusually hot and dry summer of 2018, have highlighted the potential for sudden shifts in tick distribution [202]. That year, *H. marginatum* was reported in Austria [31], Western Germany [36], and Sweden [26], while *H. rufipes* was also detected in Germany and the United Kingdom [36, 156]. These findings likely resulted from the molting of nymphs introduced by migratory birds under favorable climatic conditions. Understanding the locations of migratory bird stopovers and resting sites is, therefore, critical for predicting new tick populations and the use of citizen science and bird migration data may significantly enhance predictive model performance [203].

In addition to climate and avian-mediated dispersal, the availability of suitable hosts is essential for population establishment. Adult *H. marginatum* preferentially feed on large mammals and the absence of such hosts can limit population sustainability [204]. Moreover, a critical density of molting individuals must be reached to establish a reproducing population capable of surviving seasonal environmental fluctuations [205].

Socioeconomic conditions, agricultural practices, and human-induced land use changes also significantly influence tick distribution [179, 206]. Globalization has accelerated the spread of exotic vectors and pathogens through increased trade and travel [207]. In addition to these factors, arrmed conflicts and climate-related displacement may further drive tick dispersal by disrupting land use, veterinary controls, and biosecurity measures. The movement of displaced people and livestock, often without proper surveillance, increases the risk of introducing infested hosts into new regions. These dynamics highlight the need for preventive strategies that consider both climate and non-climate factors.

Predictive modeling is an essential tool for anticipating future range shifts of *H. marginatum* and guiding targeted surveillance and intervention strategies. To improve the accuracy and ecological relevance of these models, a suite of meaningful variables should be included, such as long-term temperature indices, relative humidity, seasonal precipitation, land cover, and land use changes. Additionally, data on livestock densities and the geographic distribution of domestic and wild hosts are important. Socioeconomic variables may also be important for modeling. While human population density is commonly used as a predictor in ecological models for many tick species, its relevance for *H. marginatum* specifically remains uncertain. To date, no published studies have shown whether human density enhances or detracts from model performance for this species. Therefore, both environmental and anthropogenic variables should be carefully assessed to develop accurate and evidence-based tools for surveillance and disease prevention.

This review highlights the need for strengthened tick surveillance and public health awareness, particularly in high-risk regions and during heatwave years that may favor tick survival and expansion. Integrated monitoring strategies should focus on migratory bird routes and livestock-dense areas. Future research must address gaps in our understanding of CCHFV transmission, including the vector competence of local ticks and the reservoir role of hosts. Developing affordable, sensitive diagnostics is essential for expanding surveillance in resource-limited settings. Finally, a standardized surveillance framework across Europe is crucial to improve data comparability, predictive modeling, and preparedness in the context of climate change and increasing animal movement.

## 7. Conclusion

*Hyalomma marginatum* has increasingly been detected beyond its native range, raising significant public health concerns. This review highlights that the spread of *H. marginatum* ticks into nonnative regions is primarily facilitated by migratory birds and animal trade, with these imported ticks often testing positive for pathogens such as CCHFV and *Rickettsia* species. As a primary vector for CCHFV and a major vector of SFGR, the expansion of *H. marginatum* in Europe could substantially heighten public health risks, particularly under the influence of climate change.

To mitigate these risks, proactive monitoring of *H. marginatum* spread is essential, especially in areas where it has not been previously established. Monitoring should encompass surveillance of migratory bird routes, identification of favorable tick habitats, and analysis of environmental factors supporting tick establishment. These insights will be crucial for developing control strategies aimed at reducing the risk of CCHFV and other *Hyalomma*-borne pathogens impacting public health and livestock in Europe.

While climate change is expected to significantly affect *H. marginatum* distribution, additional factors such as socioeconomic development, agricultural practices, and land use changes may also drive shifts in its range. Therefore, preventive measures should address both climate-related and non-climate factors to effectively limit the introduction and establishment of exotic ticks and associated pathogens in new regions.

## Figures and Tables

**Table 1:** Pathogens detected in *Hyalomma marginatum* in Europe and its associated diseases.

Pathogen group	Etiological agent	Disease
Bacteria		
Anaplasma	*Anaplasma* spp.	Anaplasmosis
	*Anaplasma phagocytophilum*	Human granulocytic anaplasmosis, ovine anaplasmosis
	*Anaplasma platys*	Thrombocytic anaplasmosis
	*Anaplasma marginale*	Bovine anaplasmosis
Bartonella	*Bartonella* spp.	Bartonellosis
Borrelia	*Borrelia burgdorferi* s.l.	Lyme borreliosis
	*Borrelia lusitaniae*	Lyme borreliosis
Coxiella	*Coxiella burnetii* *Ehrlichia* spp.	Q fever Ehrlichiosis
Ehrlichia	*Ehrlichia minasensis*	Ehrlichiosis
Francisella	*Francisella*-like	Francisellosis
Rickettsia	*Rickettsia* spp.	Tick-borne rickettsiosis
	*Rickettsia sibirica mongolitimonae*	Lymphagitis-associated rickettsiosis (LAR)
	*Rickettsia aeschlimannii*	Tick-borne lymphadenopathy (TIBOLA)
	*Rickettsia africae* *Rickettsia raoultii*	African tick-bite fever Tick-borne lymphadenopathy (TIBOLA)
Protozoa		
Babesia	*Babesia bigemina*	Bovine babesiosis (Texas cattle fever)
	*Babesia bovis*	Bovine babesiosis
	*Babesia caballi*	Equine piroplasmosis
	*Babesia occultans* *Theileria* spp.	Bovine piroplasmosis Theileriosis
Theileria	*Theileria annulata*	Tropical theileriosis (Mediterranean bovine theileriosis)
	*Theileria equi*	Equine piroplasmosis
	*Theileria buffeli/sergentii/orientalis*	Bovine theileriosis (Theileria-associated bovine anemia)
Viruses		
	Crimean–Congo hemorrhagic fever virus	Crimean–Congo hemorrhagic fever
	West Nile virus	West Nile encephalitis
	Dhori virus	Dhori virus infection
	Parapoxvirus	Orf (Contagious ecthyma) Bovine papular stomatitis Pseudocowpox

**Table 2:** Summary of the key findings of published studies on the prevalence of Crimean–Congo hemorrhagic fever virus (CCHFV) in *Hyalomma marginatum* ticks across Europe.

Country	Province	Collection date	Number of tested/collected *H. marginatum* ticks	Prevalence of CCHFV	Stage of CCHFV positive tick^a^	Source	Method of detection	Reference
Albania	Kukes	2007–2014	—	4.7% (16/341)	A	Livestock	Nested RT-PCR	[48]
Bosnia	—	2019–2021	122/122	2.5% (3/122)	A	Livestock	qRT-PCR	[68]

Bulgaria	Region of Stara Zagora, Central Bulgaria	May–June, 2006–2010	—	3.7% (6/161)	A	Livestock	IFHA, RT-PCR, RT-Nested-PCR	[55]
	Region of Haskovo, Southeastern Bulgaria	May–June, 2006–2010	—	13.9% (5/36)	A	Livestock	IFHA, RT-PCR, RT-Nested-PCR	—
	Region of Kardzhali, Southeastern Bulgaria	May-June, 2006–2010	—	3.4% (3/87)	A	Livestock	IFHA, RT-PCR, RT-nested-PCR	—
	Kardzhali	June–July, 2014	233/389	8.6% (20/233)	A	Livestock	qRT-PCR	[151]
	Burgas	June–July, 2014	217/388	8.8% (19/217)	A	Livestock	qRT-PCR	—

Kosovo	Prizren	April–June, September–October, 2014–2018	—	11% (1/9)	A (M)	Livestock	qRT-PCR	[54]
	Malishevë	May–June, 2012	148/244	14.2% (21/148)	A	Cattle, goat	qRT-PCR	—
	Klinë	May–June, 2012	119/167	5.9% (7/119)	A	Cattle, goat	qRT-PCR	[58]
	Gjilan	May–June, 2012	30/156	3.3% (1/30)	A	Cattle	qRT-PCR	—
	Stavropol, Volgograd, Astrakhan, and Rostov	2000	4787/4787	10.2 % (46 of 449 pools)	na	na	ELISA	[152]

Russia	Stavropol, Rostov, Krasnodar, Dagestan,Karachay-Cherkess Republic, Crimea	2012–2019	2045/10,257A; 285/15,536L-N	7.2% (148/2045A); 21% (60/285L-N)	L/N/A	Cattle, sheep, horse, human, European hare, hedgehog, rook, crested lark	Nested-PCR	[153]

Spain	Cáceres	2015	—	20% (1/5)	A (F)	Cattle	RT-PCR	[99]

^a^A, adult tick; M, adult male tick; F, female adult tick.

**Table 3:** Summary of the key findings of published studies on the prevalence of *Rickettsia* species in *Hyalomma marginatum* ticks across Europe.

Country	Province	Collection date	Species of *Rickettsia*	Prevalence of *Rickettsia* species	Stage of *Rickettsia* positive ticks^a^	Source	Method of detection	Reference
Austria	Melk, Lower Austria	2 October, 2018	*R. aeschlimannii*	%100 (1/1)	A (M)	Horse	qPCR	[31]

Croatia	Kastela Bay, Split, Dalmatia County	October, 2000	*R. aeschlimannii*	64.7% (11/17)	na	Cattle	PCR	[61]

France	Corsica	May–May, 2014–2015	*R. aeschlimannii*	%100 (89 pools/89 pools - 362 ticks)	A	—	PCR, qPCR	[29]
	Ponte-Leccia slaughterhouse, Corsica	May–September, 2016	*Rickettsia* spp.	59% (16 pools/27 pools)	A	Cattle	PCR, qPCR	[74]
	Ponte-Leccia slaughterhouse, Corsica	May–August, 2017; July–December, 2018	*R. aeschlimannii*	83.5% (81 pools/97 pools - 216 ticks)	na	Cattle	PCR, qPCR	[28]
	Corsica	August–January, 2018–2019	*R. aeschlimannii*	%100 (1/1)	A (F)	Wildboar	PCR, qPCR	[28]
	Corsica	2018–2020	*R. aeschlimannii*	50% (1/2)	A	Wildboar	PCR, qPCR, nested-PCR	[79]
	Corsica	2019–2021	*R*. *aeschlimannii*	%100 (23/23)	na	Cattle	PCR, qPCR	[80]

Germany	Wächtersbach, Hesse	26 June, 2018	*R. aeschlimannii*	%100 (1/1)	A (F)	Sheep	PCR, qPCR, qRT-PCR	[36]
	Wächtersbach, Hesse	5 August, 2018	*R. aeschlimannii*	%100 (1/1)	A (M)	Horse	PCR, qPCR, qRT-PCR	
	Hannover, Hesse	24 August, 2018	*R. aeschlimannii*	%100 (1/1)	A (M)	Car	PCR, qPCR, qRT-PCR	
	Neuenhaus, Lower Saxony	22 August, 2018	*R. aeschlimannii*	%100 (1/1)	A (F)	Horse	PCR, qPCR, qRT-PCR	
	Fechenheimer Aue, Hesse	4 September, 2018	*R. aeschlimannii*	%100 (1/1)	A (F)	House	PCR, qPCR, qRT-PCR	
	Saxony-Anhalt	May, 2007	*R. aeschlimannii*	%100 (3/3)	A	Eurasian reed warbler	PCR	[35]

Greece	Northern Greece	June, July, September, December, 2013	*Rickettsia* spp.	%100 (1/1)	N	Eastern olivaceous warbler	Nested-PCR	[63]

Hungary	Ocsa Ringing Station, Duna-Ipoly National Park	2011	*Rickettsia* spp.	66.6% (2/3)	L (N)	European robin	PCR	[37]

Italy	Ogliastra and Sassari, Sardinia	June–July, 2007	*R. aeschlimannii*	%100 (11/11)	A	Horse	PCR	[140]
	Corleone, Palermo	—	*R. aeschlimannii*	4.2% (2/48)	na	Livestock	PCR	[142]
	Corleone, Palermo	—	*R. africae*	2.1% (1/48)	na	Livestock	PCR	
	Sicily	—	*Rickettsia* spp.	4.5% (3/67)	na	Cattle	PCR, reverse lineblot	[127]
	Pianosa	2004–2007	*R. aeschlimannii*	18.2 (2/11)	na	Ground	PCR	[124]
	Urzulei, Ogliastra	10 June, 2010	*R. aeschlimannii*	8.3% (1/12)	A	Horse	PCR	[115]
	Castel di Guido and Rome	July–September, 2010–2011	*R. aeschlimannii*	21.1% (8/38)	N	Migratory birds	PCR, qPCR	[27]
	Ponzian Islands and Rome	April–May, 2010–2011	*R. aeschlimannii*	50.7% (36/71)	N	Migratory birds	PCR, qPCR	
	Livorno, Pianosa Island	2012–2013	*R. aeschlimannii*	10% (1/10)	na	Ground	Nested-PCR	[121]
	Messina, Sicily	2012–2013	*R. aeschlimannii*	%100 (2/2)	na	Human	PCR	[119]
	Gairo, Sardinia	May–September, 2014	*R. aeschlimannii*	50% (1/2)	na	Mouflon	PCR	[125]
	Sardinia	2010–2015	*Rickettsia* spp.	33% (15/45)	10F/3M/2N	Bird, goat, hedhehog, horse, human, sheep	PCR	[133]
	Uta, Sardinia	March–November, 2017	*R. aeschlimannii*	28.6 (2/7)	N/A	Cattle	—	[154]
	Monte Romano, Viterbo	2012–2013	*R. aeschlimannii*	66% (8/12)	na	Ground	PCR, qPCR	[118]
	Ventotene island	April–May, 2013	*R. aeschlimannii*	50% (5/10)	N	Migratory birds	qPCR	[122]

Italy–Greece	Capri (Italy), Antikythira (Greece)	2009–2010	*Rickettsia* spp.	2.4% (16/658)	7L/9N	Migratory birds	qPCR	[24]
	Capri (Italy), Antikythira (Greece)	2009–2010	*R. aeschlimannii*	45.7% (301/658)	73L/228N	Migratory birds	qPCR	
	Capri (Italy), Antikythira (Greece)	2009–2010	*R. africae*	2.3% (15/658)	11L/4N	Migratory birds	qPCR	

Netherlands	Odoorn, Drenthe	17 July, 2019	*R. aeschlimannii*	%100 (1/1)	A (F)	Horse	PCR, qPCR	[39]

Portugal	Bird Rehabilitation Centers of Monsanto Forest Park; Quercus Santo Andre	2002–2004	*R. aeschlimannii*	6.7% (1/15)	N	Common kingfisher	PCR	[92]
	Bird Rehabilitation Centers of Monsanto Forest Park; Quercus Santo Andre	2002–2004	*R. aeschlimannii*	33% (1/3)	N	Little owl	PCR	
	Bird Rehabilitation Centers of Monsanto Forest Park; Quercus Santo Andre	2002–2004	*R. aeschlimannii*	25% (4/3)	N	Eurasian eagle-owl	PCR	
	Beja	December–September, 2013–2015	*R. aeschlimannii*	12.1% (13/107)	10M/3F	Cattle	PCR, nested-PCR	[25]
	Beja	December–September, 2013–2015	*R. raoultii*	5.6% (6/107)	1M/5F	Cattle	PCR, nested-PCR	
	Faro and Beja	December–September, 2013–2015	*R. aeschlimannii*	5.3% (1/19)	N	Little owl	PCR, nested-PCR	

Russia	Stavropol	2004	*R. aeschlimannii*	20% (8/40)	20F/20M	Cattle, vegetation	PCR	[88]
	Kaliningrad	2009	*R. aeschlimannii*	20% (1/5)	1L/4N	Tree pipit, common redstart, common blackbird	PCR	[89]

Spain	La Rioja	2001–2005	*R. aeschlimannii*	40% (2/5)	na	Human	PCR, seminested-PCR	[106]
	La Rioja	2001–2005	*R. aeschlimannii*	0.9% (1/110)	na	Cattle	PCR, seminested-PCR	
	Central Spain, Madrid and Toledo	March–December, 1999–2002	*R. aeschlimannii*	61.5% (8/13)	na	Livestock, birds	PCR	[111]
	La Rioja	2012–2015	*R. aeschlimannii*	16.7% (2/12)	A	Human	PCR, qPCR	[23]
	La Rioja	2009–2015	*R. aeschlimannii*	%100 (7/7)	N	Great reed warbler	PCR, qPCR	
	La Rioja	2009–2015	*R. aeschlimannii*	40% (4/10)	N	Eurasian reed warbler	PCR, qPCR	
	La Rioja	2009–2015	*R. aeschlimannii*	16.7% (1/6)	N	Eurasian blue tit	PCR, qPCR	
	La Rioja	2009–2015	*R. aeschlimannii*	33.3% (6/18)	2L/4N	Ortolan bunting	PCR, qPCR	
	La Rioja	2009–2015	*R. aeschlimannii*	75% (6/8)	L	Woodlark	PCR, qPCR	
	La Rioja	2009–2015	*R. sibirica mongolitimonae*	10% (1/10)	A (F)	Common nightingale	PCR, qPCR	
	La Rioja	2009–2015	*R. aeschlimannii*	30% (3/10)	3 N	Common nightingale	PCR, qPCR	
	La Rioja	2009–2015	*R. aeschlimannii*	50% (1/2)	N	Eurasian golden oriole	PCR, qPCR	
	La Rioja	2009–2015	*R. sibirica mongolitimonae*	12.5% (1/8)	A (M)	Great tit	PCR, qPCR	
	La Rioja	2009–2015	*R. aeschlimannii*	25% (2/8)	1L/1M	Great tit	PCR, qPCR	
	La Rioja	2009–2015	*R. aeschlimannii*	50% (1/2)	L	Rock sparrow	PCR, qPCR	
	La Rioja	2009–2015	*R. aeschlimannii*	6.5% (2/31)	1L/1N	Common blackbird	PCR, qPCR	
	Palencia	2013	*R. aeschlimannii*	54% (13/24)	N	Common buzzard	PCR, qPCR	
	Castilla y León	na	*R. aeschlimannii*	5.9% (19/324)	na	Human	PCR	[100]
	Castilla y León	1997–2003	*R. aeschlimannii*	6.1% (26/426)	na	Human	PCR	[101]
	Castilla y León	2004–2007	*R. aeschlimannii*	14.2% (73/515)	46M/26F/1N	Human	PCR	[101]

Sweden	Nyköping, Södermanland	21 August, 2018	*R. aeschlimannii*	%100 (1/1)	A (F)	Horse	qRT-PCR	[26]
	Upplands Väsby, Stockholm	5 September, 2018	*R. aeschlimannii*	%100 (1/1)	A (F)	Horse	qRT-PCR	[26]
	Funnarp, Hästveda, Hässleholm, Skåne	25 September, 2018	*R. aeschlimannii*	%100 (1/1)	A (M)	Cattle	qRT-PCR	[26]
	Katthammarsvik, Gotland	11 October, 2018	*Rickettsia* spp.	%100 (1/1)	A (F)	Horse	qRT-PCR	[26]
	Ovanåker, Hälsingland	18 September, 2018	*R. aeschlimannii*	%100 (1/1)	A (M)	Horse	qRT-PCR	[26]

UK	East Anglia, England	21 June, 2018	*R. aeschlimannii*	%100 (1/1)	A	Human	qPCR	[82]

Prevalence of *Rickettsia* species in non-*Hyalomma marginatum* ticks across Europe								

Italy, Greece^c,d^	Capri and Antikythira	2009–2010	*R. aeschlimannii*	50.3% (331/658)	L/N	Migratory birds	qPCR	[24] —
				%100 (1/1)	N			

Italy^b^	Ponzian Islands and Rome	2010–2011	*R. aeschlimannii*	50.7% (36/71)	N	Migratory birds	PCR, qPCR	[27]

Italy^b^	Ponza Island	March–May, 2019	*R. aeschlimannii*	%100 (1/1)	N	Common whitethroat	qPCR	[155]
						Northern wheatear		
						Whinchat		

Germany^b^	Hannover, Lower Saxony	August, 2018	*R. aeschlimannii*	%100 (1/1)	A	Horse	PCR, qPCR, qRT-PCR	[36]
	Saulheim, Rhineland-Palatinate							
	Neuenkirchen, Lower Saxony	October, 2018						
	Mörsdorf, Rhineland-Palatinate							

Sweden^b^	Klintehamn	2018	*R. aeschlimannii*	%100 (1/1)	A (M)	Horse	qRT-PCR	[26]
	Örebro				A (F)			
	Älvnäs, Vålberg				A (M)			
	Sölvesborg							
	Vreta kloster				A (F)			
	Örserum, Gränna				A (M)			
	Bjurbäcken							

UK^b^	Dorset, England	September, 2018	*R. aeschlimannii*	%100 (1/1)	A	Horse	PCR, qPCR	[156]

^a^A, adult tick; M, adult male tick; F, female adult tick; N, nymph; L, larva.

^b^ *Hyalomma rufipes*.

^c^ *Hyalomma marginatum* sensu lato.

^d^ *Hyalomma marginatum* sensu stricto.

## Data Availability

The data that support the findings of this study are available in the supporting information of this article.

## Supporting Information

Supporting Information Detailed dataset presenting all records of *Hyalomma marginatum* distribution, associated hosts, and detected pathogens across Europe, compiled and analyzed for this systematic review.
